# Group VA Aromatic Thiosemicarbazone Complexes: Synthesis, Characterization, Biological Activity, and Topological Studies

**DOI:** 10.3390/ijms251910794

**Published:** 2024-10-08

**Authors:** Ibrahim I. Ozturk, Emine I. Sumer, Grzegorz Dutkiewicz, Christina N. Banti, Sotiris K. Hadjikakou, Anita M. Grześkiewicz, Maciej Kubicki

**Affiliations:** 1 Section of Inorganic Chemistry, Department of Chemistry, Tekirdag Namık Kemal University, 59030 Tekirdag, Türkiye; iiozturk@nku.edu.tr (I.I.O.);; 2 Ethnochem Limited Company, Silahtaraga, University 1st Street, 13/1, Z102, 59860 Tekirdağ, Türkiye; 3 Department of Chemistry, Adam Mickiewicz University, Uniwersytetu Poznanskiego 8, 61-614 Poznan, Poland; 4 Department of Chemistry, University of Ioannina, Biological Inorganic Chemistry Lab, 45110 Ioannina, Greece; cbanti@uoi.gr (C.N.B.); shadjika@uoi.gr (S.K.H.); 5 University Research Center of Ioannina (URCI), Institute of Materials Science and Computing, 45110 Ioannina, Greece

**Keywords:** antimony (III), bismuth (III), aromatic thiosemicarbazones, crystal structure, biological studies

## Abstract

The antiproliferative and antibacterial activities of thiosemicarbazones increase markedly with the presence of metal ions. One of the factors determining the activity of metal thiosemicarbazone complexes is the coordination structure. In this study, the biological effects of new antimony (III) and bismuth (III) thiosemicarbazone complexes with different binding modes and geometrical structures were demonstrated. Three new complexes, with the formulae {[SbCl_3_(µ_2_-S-Hacptsc)(η^1^-S-Hacptsc)], 2/3H_2_O,1/3CH_2_Cl_2_}, {[SbCl_3_(κ^2^-S,N-Hacpmtsc)(η^1^-S-Hacpmtsc)_2_CH_2_Cl_2_]}, and{[BiCl_3_(η^1^-S-Hbzmtsc)_3_]·C_2_H_5_OH}, where Hacptsc: acetophenone thiosemicarbazone, Hacpmtsc: acetophenone-N-methyl thiosemicarbazone, Hbzmtsc: benzaldehyde-N-methyl thiosemicarbazone) were elucidated by different methods and deeply analyzed in accordance with their structure by X-ray structure analysis and Atoms-In-Molecules topological analysis. This analysis provided a deeper understanding of the coordination spheres of the Sb/Bi complexes. For instance, the first reported two binding modes of the same ligand are observed in a single crystal structure of antimony (III) halide complexes. Additionally, in one of the complexes, a solid-to-solid phase transition was detected and analyzed in detail. Those complexes, very unique in terms of their geometry, have also been tested for their in vitro cytotoxic activity against human adenocarcinoma cervical cancer (HeLa) cells, whereas antimony (III) complex 1is the most active complex of this study. Further, the antibacterial activity of the complexes has been screened against two Gram-negative (*Pseudomonas aeruginosa* and *Escherichia coli*) and two Gram-positive (*Staphylococcus epidermidis* and *Staphylococcus aureus*) pathogenic bacteria. From the results, it is found that all the complexes exhibited significant activity against the Gram-negative pathogenic bacteria.

## 1. Introduction

The use of metals and metal-containing compounds in medicine dates back to ancient times. The earliest records of using antimony for medicinal purposes can be traced to the Assyrians, who used it primarily to treat urinary diseases, although its main application was as an eye ointment for cosmetic purposes. In medieval Europe, antimony was employed in a variety of medical treatments despite the recurring reports of its toxic effects. Notably, its ability to “cleanse” patients by inducing sweating, vomiting, and purging was considered an acceptable alternative to bloodletting [1,2]. Antimony and its compounds (e.g., tartrate and potassium tartrate) were also recommended for the treatment of syphilis, melancholy, chest pains, fevers, and other ailments. The popularity of antimony-based drugs began to decline in the 19th century, with a brief resurgence for the treatment of sleeping sickness, as certain antimony compounds were found to be effective against trypanosomes. Today, antimony is mainly used in industrial applications. It is worth noting that antimony has been included in the list of critical raw materials (latest list [3]).

In contrast, the history of bismuth is quite different. Bismuth salts were used as early as the 18th century to treat syphilis and skin lesions. Bismuth compounds exhibit antimicrobial activities against both Gram-positive and Gram-negative bacterial pathogens, and they are known for their potential in combating multi-drug-resistant infections (e.g., [4,5,6]). Today, the primary medical use of bismuth-based drugs is to eradicate *Helicobacter pylori*, a Gram-negative bacterium that causes peptic ulcers and other gastrointestinal diseases.

It is important to note that the activity and toxicity of bismuth- and antimony-based compounds depend on various factors, such as the type of ligand, the coordination structure of the complex, solubility, stability, oxidation states, and more [4,7]. Therefore, detailed studies of the interactions between Sb/Bi and ligands, or with other Sb/Bi centers, are crucial for advancing knowledge that could be applied in drug discovery.

Thiosemicarbazones are a particularly interesting class of ligands due to their extensive pharmacological properties, including antibacterial, antiviral, antifungal, antimicrobial, and anticancer activities. These ligands form stable complexes with metals (e.g., [8,9,10,11]). However, the structural chemistry of the complexes formed by aromatic thiosemicarbazones with main group elements is still limited

**Scheme 1:**
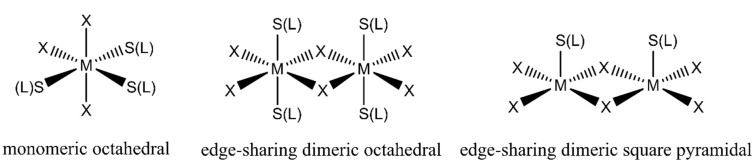
Possible coordination modes of the main group–aromatic thiosemicarbazone complexes (M: Sb or Bi; X: Cl, Br, or I; and S(L): aromatic thiosemicarbazones).

[12,13,14,15]. Depending on the molar stoichiometric ratio (e.g., 1:1, 1:2, and 1:3 metal), these main group–aromatic thiosemicarbazone complexes can form different coordination structures (Scheme 1).

**Scheme 2:**
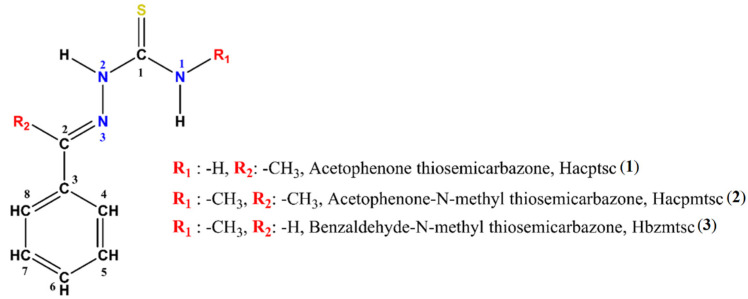
The molecular formulae of the aromatic thiosemicarbazone used in this study.

In this study, we prepared two antimony (III) and one bismuth (III) chloride complexes of aromatic thiosemicarbazones (Scheme 2) and investigated their spectroscopic properties, thermal stability, and cytotoxic and antibacterial activities. However, our primary focus—building on previous findings—was on the single-crystal structure analysis of these complexes. A detailed analysis, including a topological analysis using the Atoms-in-Molecules methodology [16], was conducted to scrutinize the coordination sphere of these group 5A (pnictogen) atom representatives.

## 2. Results and Discussion

**Figure 1:**
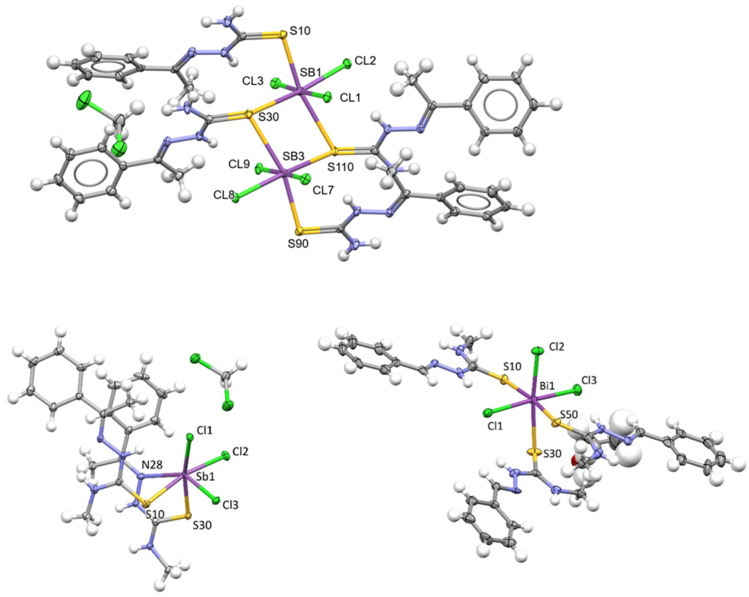
The perspective views of dimer B of complex **1** (**α**) (**top**), complex **2** (**bottom left**), and complex **3** (**bottom right**), the ellipsoids are drawn at the 50% probability level, hydrogen atoms are shown as spheres of arbitrary radii. The following colours have been assigned to the atoms: grey-carbon, white-hydrogen, blue-nitrogen, red-oxygen, green-chlorine, violet-bismuth/antimony atom.

Three new complexes with the formulae {[SbCl_3_(µ_2_-S-Hacptsc)(η^1^-S-Haptsc)]_2_ C_2_H_5_OH, 2H_2_O, CH_2_Cl_2_} (**1**), {[SbCl_3_(κ^2^-S,N-Hacpmtsc)(η^1^-S-Hacpmtsc)_2_, CH_2_Cl_2_]} (**2**), and {[BiCl_3_(η^1^-S-Hbzmtsc)_3_]·C_2_H_5_OH} (**3**) have been obtained and characterized, the ligands are shown schematically in Scheme 2, and the complexes, as observed in their crystal structures, in Figure 1.

In all the complexes, the metal/metalloid (For the simplification and less cumbersome reading, in the rest of the article we will use only the term “metal” also for antimony, when the analysis will be related to both. However, it should be remembered that antimony has properties intermediate between metals and non-metals) centers are more or less deformed and octahedrally coordinated by three chlorides and different combinations of the atoms from the ligands (S_3_ or S_2_N), even though the ligands are very similar.

### 2.1. Structural Analysis

Complex **1** is a binuclear, edge-sharing antimony (III) complex with a ligand–antimony ratio of 4:2 (Figure 1). Each ligand coordinates to the antimony ion in its neutral thione form, acting as a monodentate donor via sulfur atoms, with two ligands bridging. Edge-sharing binuclear main group metal complexes with octahedral geometry around the central atom can adopt four different isomeric forms [17]. In complex **1**, the monomeric units are connected by sulfur bridges, resulting in the rarest of these isomeric forms: a sulfur-bridged, edge-sharing binuclear structure. Among all the binuclear complexes of Bi and Sb deposited in the Cambridge Structural Database (CSD [18]) that contain both halogen and sulfur substituents, this form is observed in less than 6% of the cases.

**Figure 2:**
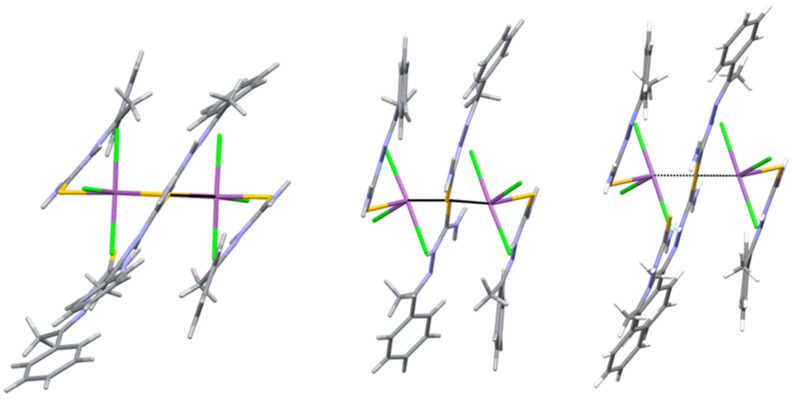
The perspective views of the dimers observed in the crystal structures of **1**, as seen perpendicularly the bridging plane: from the **α** phase symmetrical dimer A (**left**), and unsymmetrical dimer B (**middle**) and symmetrical dimer C from the **β** phase (**right**). The following colours have been assigned to the atoms: grey-carbon, white-hydrogen, blue-nitrogen, red-oxygen, green-chlorine, violet-bismuth/antimony atom.

Even more intriguing from a structural standpoint, complex **1** exhibits a reversible solid-to-solid phase transition. In the temperature range of −173 °C to +27 °C, two phases were identified: a low-temperature **α** phase and a room-temperature **β** phase. Both crystallize in the triclinic *P-1* space group but with different unit cell parameters In the **β** phase, the asymmetric unit contains only half of a symmetry-independent molecule (a *Ci*-symmetrical dimer, C). In the **α** phase, however, there are three independent monomers forming two dimers: A, which is also *Ci*-symmetrical, and B, an unsymmetrical dimer. These two dimers exhibit significantly different geometries (Figure 2).

**Figure 3:**
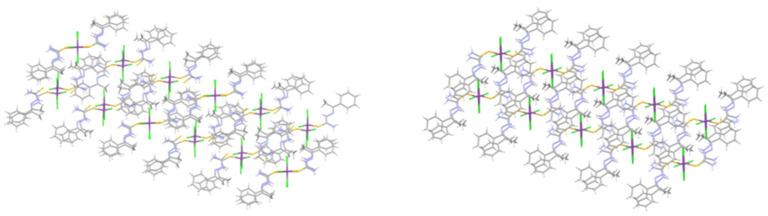
The crystal packing schemes of two phases of complex **1** (**α** phase (**left**) and **β** phase (**right**)), as seen perpendicular to the N11-H11b···Cl3/N111-H11c···Cl6 (hydrogen bond chains). The following colours have been assigned to the atoms: grey-carbon, white-hydrogen, blue-nitrogen, red-oxygen, green-chlorine, violet-bismuth/antimony atom.

The transformation between the two phases is reversible and occurs in a single crystal, meaning that the structural changes cannot be drastic, and the overall similarity of the molecules should be maintained. This raises the question: what exactly changes, and why? An analysis of the crystal packing at −173 °C (**α** phase) and room temperature (**β** phase) reveals that in both cases, the main structural elements are the N-H···Cl and N-H···S hydrogen bonds. The presence of relatively large voids, filled with disordered solvents, allows for geometric changes and shifts in the positions of the monomers. These shifts ultimately lead to symmetry changes without altering the hydrogen bonding pattern (see Figure 3).

**Figure 4:**
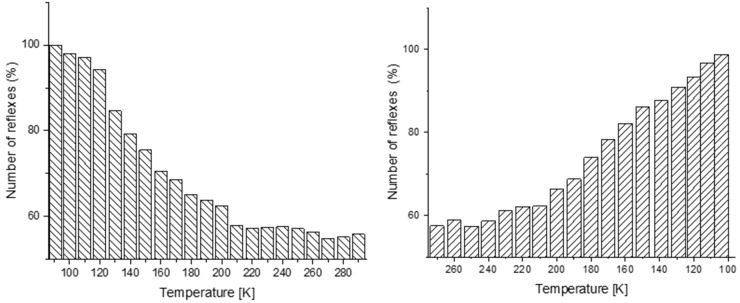
The number of the reflections at different temperatures during increasing (**left**) and lowering (**right**) the temperature.

This transformation results in changing the void area in the crystal structure and stabilizing the position of one of the disordered solvent molecules (dichloromethane), which in the **α** form can be reasonably modeled, while in the **β** form, it cannot. The void area decreases from 10.5% of the unit cell to 8.8% (Probe radius 1.2 Å, approximately grid sparing 0.3 Å) (before the modeling of the dichloromethane). The phase transition is continuous rather than discrete; with lowering the temperature, the share of **α** form increases (Figure 4). The analysis of the graph in Figure 4 shows that this transformation starts somewhere around −73 °C; in this range, we see the most significant increase in the number of observed reflections. Moreover, it seems to be the initial temperature while both increasing and lowering the temperature. The first reflections from the **α** phase at −73 °C are very weak: at −153 °C, the intensities of the reflections from the **α** form are around ¼ of those from the **β** form, while at −173 °C, this proportion is close to ½. That indicates that probably in the measured ranges of temperature, the phase transition is still in process.

**Figure 5:**
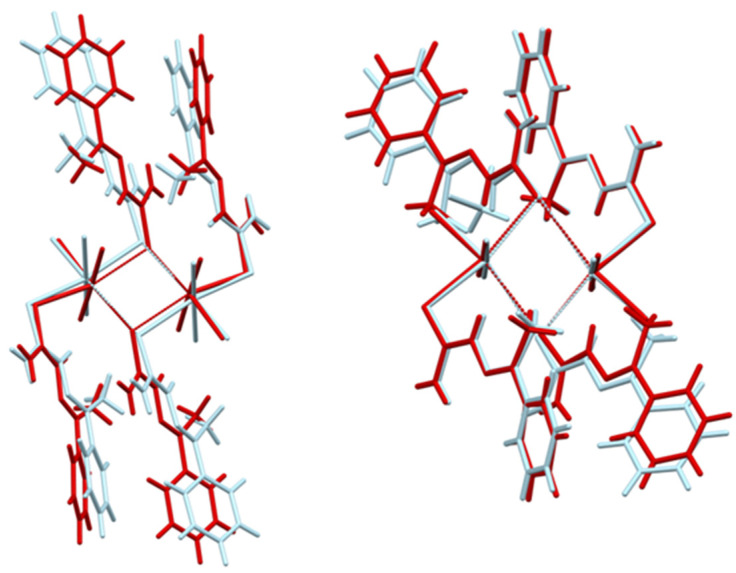
The overlay of the dimers A (**left**) and B (**right**) from the **α** phase (light blue) with the dimer from the **β** phase (red).

The geometry of dimer C observed in the **β** phase more closely resembles the unsymmetrical B dimer than the symmetrical A form in the **α** phase (Figure 2 and Figure 5).

In all the dimers, the geometry around the metalloid center is close to the octahedral one. However, in dimer A (where the inversion center lies in the middle of the Sb2···Sb2 (*-x*, *-y*, *-z*) distance), the deformation of the octahedron is much smaller than for the B, C dimers (cf. Table S1 Supplementary Materials). The largest deviation from 180° for the angles between the opposite coordination centers in dimer A is smaller than 10° (S50-Sb2-S70170.48(3)°), while for dimer B, this value is close to 30°, and for two angles (Cl2-Sb1-S30 is 155.53(3)° and Cl8-Sb3-S110 152.79(3)°). Similar values can be observed in C, where the Cl2-Sb1-S30 angle is 159.46(2)°. The consequence of the more disordered geometry can be seen in the shorter distance between the metalloid centers: for dimer A, it is 4.4414(5) Å, while for C—4.0880(2) Å and for B—3.9183(4) Å. The van der Waals radii for Sn is 2.06 Å, so the Sb···Sb distance in B is significantly, almost by 0.2 Å, shorter than the sum of van der Walls radii, while in C, it is close to this value. Such a situation is quite uncommon; for all the Sb complexes including at least one halogen ligand deposited in the CSD, it is observed only in 1.62% (54 out of 2966) structures, so it is definitely not the preferred geometry. When the database search is limited to complexes with ligands containing both sulfur and halogen donors, the percentage of those with short Sb···Sb contacts is slightly larger 3.72% (6 out of 161), but still, it is marginal.

In the mononuclear complex **2**, the antimony (III) cation coordinates with two acetophenone-*N*-methyl-thiosemicarbazone ligands and three chloride anions with distorted octahedral geometry around the antimony ion. Both of these ligands have protonated forms and one of the ligands has Z-conformation, while the other has E-conformation. The ligand with the E-conformation binds to the central atom only through the sulfur donor atom, whereas the ligand with the Z-conformation bidentate coordinates to the central atom through the azomethine nitrogen and sulfur atoms, forming a five-membered chelate ring. Complex **2** is the first example exhibiting such a bonding mode (η^1^-S, κ^2^-S, N) in antimony (III)—thiosemicarbazone chemistry. Analogs to complex **1** and complex **2** also crystallized with CH_2_Cl_2_ in the lattice.

The structure of complex **3** is a solvate of a complex that has been previously reported by our group [14]. The asymmetric unit consists of one bismuth atom, three chlorides, three benzaldehyde N-methyl-thiosemicarbazone ligands, and one solvent (ethanol) molecule. The neutral thiosemicarbazone ligands are monodentate coordinated to the bismuth ion through the sulfur atoms, and three sulfur and three chloride atoms form a distorted octahedral geometry with meridional conformation around the bismuth ion. The supramolecular structure of complex **3** is dominated by a three-dimensional network connected by weak intermolecular hydrogen bonds (N-H^…^Cl and N-H^…^S).

### 2.2. Topological Analysis

Critical point calculations have been performed to study the coordination sphere of the studied complexes in more detail, especially for complex **1** where the geometry seems to be quite perplexing. The Quantum Theory of Atoms in Molecules (QTAIM) enables us to study the nature of the bonds by means of the detailed analysis of the critical points (CPs) of the electron density distribution [16]. There are several approaches aiming to estimate the nature of the studied bonds. Traditionally, it has been assumed that the nature of the bonding can be recognized by the analysis of the value of the electron density in the given CP (*ρ_cp_*) and the sign of the Laplacian of the electron density at the BCP (∇^2^*ρ_cp_*)—the CPs with the negative Laplacian are being related to the electron-sharing interactions and with the positive Laplacian—to the closed-shell ones. Macchi [19] observed that such a simple rule is not sufficient when a metal atom is involved in the bonding. Consequently, he proposed that some other characteristics should be carefully investigated: the position of the critical point, the energy density (H_cp_), or the ratio *G_cp_*/*ρ_cp_*, where *G_cp_* is kinetic energy density. Bianchi et al. [20] distinguished different bonding regimes depending on the value of the absolute ratio of the potential energy density (*V*_cp_) to the kinetic energy density (*V_cp_*/*G_cp_*) at the BCP. Another often analyzed parameter proposed by Espinosa et al. [21] is interaction energy, calculated from the potential energy density E_int_ = ½*V*_cp_; however, this parameter has been established mainly for hydrogen bonds and therefore should be used with utmost caution to the Me-L bonds.

**Figure 6:**
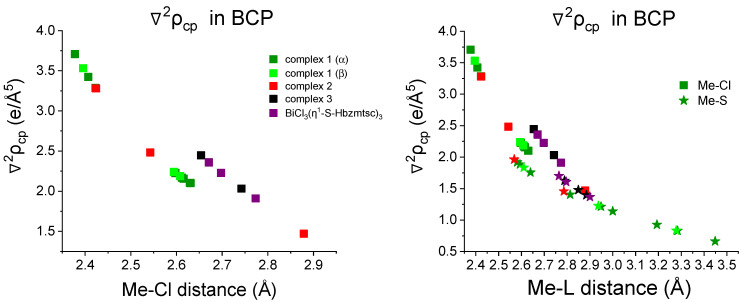
The dependence of the Laplacian of electron density on the bond length.

The topological analysis has been performed on both the polymorphic forms of complex **1**, complex **2**, and complex **3** and the analogs BiCl_3_(η^1^-S-Hbzmtsc)_3_; only CPs with electron density at the CP above 0.03 e/Å^3^ have been considered. The BCPs have been found for all the ligand-metal bonds in all three complexes (Table 1); however, their characteristics differ quite significantly, which is obviously correlated with the different bond lengths. Therefore, it seems to be more reasonable to compare them in comparison with the Me-ligand bond distance than to the absolute value of it. The analysis of the graphs in Figure 6 shows that bismuth complexes have generally higher absolute values of the Laplacian (∇^2^*ρ_cp_*), electron density (*ρ_cp_*), *G_cp_*, *V_cp_*, etc. at the BCP (cf. SM) than the antimony ones, which is rather expected. The parameters that best distinguish this difference are ∇^2^*ρ_cp_* and G*_cp_*/*ρ_cp_*, while for *H_cp_*, this disparity is barely seen. The Me-Cl and Me-S bonds can also be quite easily differentiated from Figure 6—smaller values are found for the Me-S bonds as compared to the Me-Cl ones. This observation, in the case of structures analyzed here, is true for both the antimony and bismuth complexes.

The Me-L bonds in the monomeric and dimeric antimony complexes show no systematic differences except, obviously, the characteristics of the bridging atoms, which show unquestionably weaker bonds than those in the isolated monomers. Consequently, the decreasing strength of the Me-S (bridging) bond should cause the increase in strength of the other ones in comparison with similar monomeric complex, and this is observed when we compare the CPs, for instance, of the Me-Cl atoms where the sum of E_int_ for the Sb-Cl bonds in complex **1** is about −254 kJ/mol/bohr^3^ (**α**) and −253 kJ/mol/bohr^3^ (**β**), while in complex **2**, it is −221 kJ/mol/bohr^3^.

**Figure 7:**
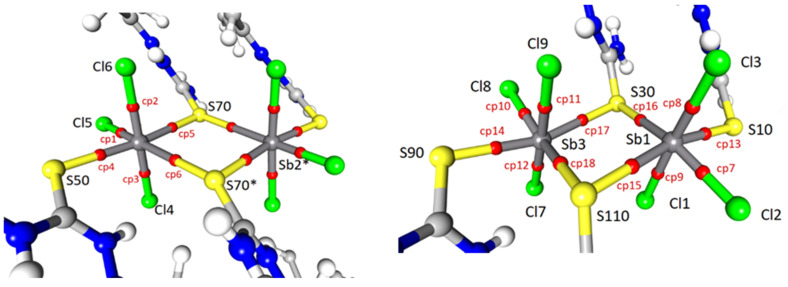
The coordination spheres of Sb centers with labeling and critical point positions. The following colours have been assigned to the atoms: light grey-carbon, white-hydrogen, blue-nitrogen, red-oxygen, green-chlorine, dark grey-antimony atom.

The topological analysis allows also us to look closer at the different dimers in complex **1** in order to—at least—suggest the source(s) of such huge differences in their geometries. A common feature of all dimers is the different lengths of the contacts between the Sb atom and bridging sulfur atoms. However, this disproportion is not equal (Figure 6, Table 1), and consequently, in dimer A the difference in distances is larger than 0.6 Å, which is reflected in the topological parameters (Table 1); for instance, H_cp_ for cp5 is −22.41 kJ/mol/bohr^3^ while for cp6 it is −0.61 kJ/mol/bohr^3^, and in the case of dimer B, the differences in distances are smaller, roughly 0.19 Å and 0.33 Å for two Sb atoms, which results in H_cp_ for cp 15 = 11.35, cp 16 = 4.82, cp17 = 13.87, and cp18 = 2.89 kJ/mol/bohr^3^ (for the numbering of CPs see Figure 7). The difference in bond lengths in the second polymorph is more similar to dimer B and equals 0.34 Å; consequently, H_cp_at cp23 = 2.98 kJ/mol/bohr^3^ while at cp24 = 14.47 kJ/mol/bohr^3^). Thus, the sulfur bridging in the dimers B and C seems to be stronger than in the A one.

**Figure 8:**
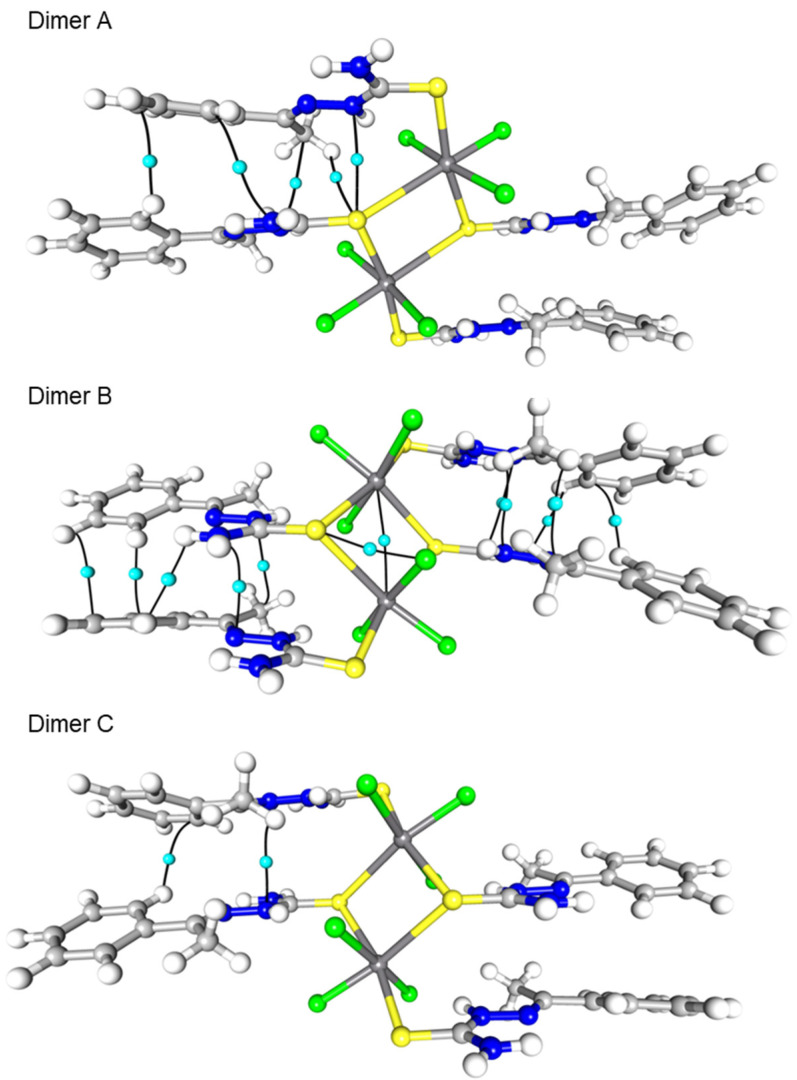
The (3, −1) non-covalent bond critical points found between the two monomeric species of upper dimer A, middle dimer B, and lower dimer C (for dimers A and C, only symmetrically independent cps are shown). The following colours have been assigned to the atoms: light grey-carbon, white-hydrogen, blue-nitrogen, red-oxygen, green-chlorine, dark grey-antimony atom.

Obviously, the dimer formation is also stabilized by the other interactions (cf. Figure 8), mostly the interactions of the delocalized electrons of both ligands.

**Figure 9:**
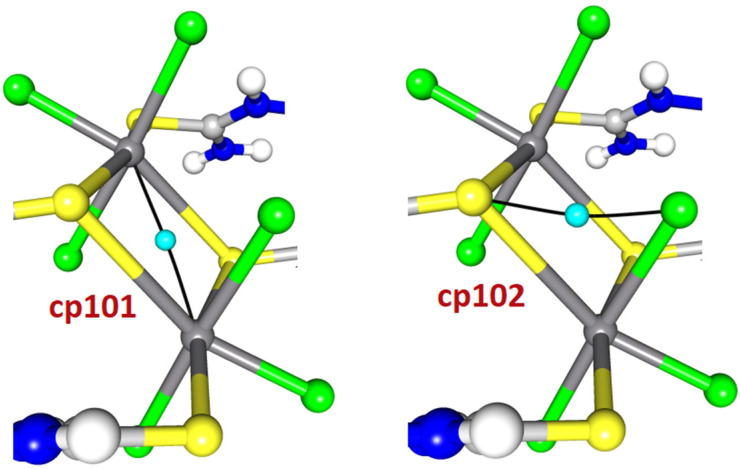
The (3, −1) non-covalent bond critical points found in dimer 2 between the Sb atoms and in the S···Cl interaction. The following colours have been assigned to the atoms: light grey-carbon, white-hydrogen, blue-nitrogen, red-oxygen, green-chlorine, dark grey-antimony atom.

The first significant topological difference between the two dimers in complex **1** in the **α** phase is the occurrence of the (3, −1) cp101 (Figure 9) and the bond path between two antimony atoms in dimer B (Figure 8 and Figure 9) which is missing in the other dimers. The characteristics of this CP meet the criteria of a “metallic bond” according to Bianchi [20]: positive Laplacian at the CP, a small negative value of the total energy density, and a relatively small value of the electron density at the critical point (as compared with those found for the Sb-Cl or Sb-S bonds). A comparison of these characteristics suggests that Sb-S(Cl) has more donor–acceptor nature (a greater value of Laplacian, negative value of total energy density, etc.) than the Sb⋅⋅⋅Sb contact.

In dimer B, one can also postulate the presence of halogen···chalcogen bonding in the core area, as suggested by the presence of the cp102 (Figure 9, Table 2). This, quite directional, interaction has the values of Laplacian and electron density at the CP similar to the weak bridging Sb-S bond. It is much stronger than any other interaction between two monomers building the dimer, but at the same time definitely weaker than the hydrogen bonds present in the structure. Interestingly, the analysis of the coordination environments of all similar structures found in the CSD (dimeric complexes with S and halogen ligands, having short Sb⋅⋅⋅Sb distance) shows that similar halogen···chalcogen interactions are present in each of them, which suggests that this type of contact (interaction) might be responsible for the stabilization of this short Sb⋅⋅⋅Sb contact. The other interactions between the “monomers” are quite similar to those found in dimer A.

In the **β** phase, despite the general geometrical similarity to dimer B, the CPs for neither Sb⋅⋅⋅Sb nor Cl⋅⋅⋅S interactions have been found.

**Figure 10:**
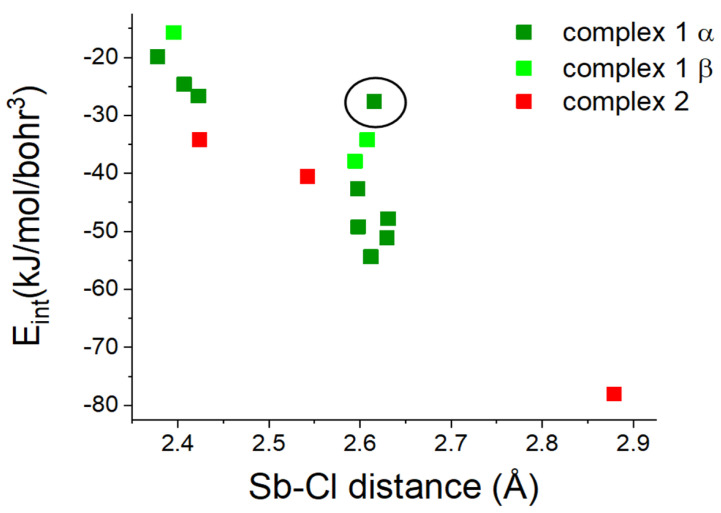
Correlation between the Sb-Cl distance and E_int_. The black oval marks the Cl1 atom from the **α** phase—see text.

As mentioned earlier, the Cl-Sb distances differ quite significantly and one of the reasons for larger or smaller elongation may be related to the interactions in which the chlorine atom is involved. Consequently, the stronger the interaction with chlorine as an acceptor, the larger bond elongation should be observed. In principle, Figure 10 shows that most atoms meet this assumption. Nevertheless, one atom (Cl1 from the **α** phase—marked with a black oval) definitely deviates from the trend defined by the others.

The Sb1-Cl1 bond distance is definitely longer than it should be judging from the interactions in which this atom is involved. However, when the environment of this atom is scrutinized, it might be realized that the Cl1 atom is quite close, actually almost at the sum of van der Waals radii limit, to the second metalloid center Sb2 (Sb2⋅⋅⋅Cl1 distance is 3.7675(9) Å). If then the Sb1-Cl1 distance is shorter, the electrons from both atoms would share the common space, obviously causing repulsion, which may be the reason for the longer-than-expected Sb-Cl bond elongation.

**Figure 11:**
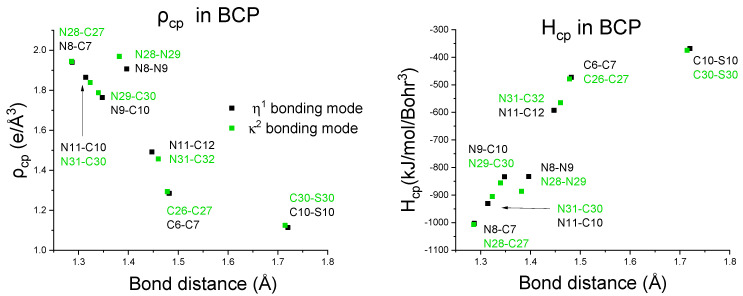
Correlation between *ρ_cp_*, *H_cp_* and the Sb-Cl distance [Å].

The other interesting issue—as has been mentioned before—is the coordination mode in complex **2**; the η^1^-S, κ^2^-S, N bonding mode allows us to compare two different types of chelating in one complex. In the case of each mode, the (3, −1) CP has been found; however, their characteristics for the Sb-N bond/contact indicate rather weak interaction between those atoms. First of all, quite a long distance between Sb and N28, 2.817(8) Å, and the low value of Laplacian at the CP (1.38 e/Å^5^) points to rather minor contribution in donating electrons in comparison to the other ligands; however, such a contribution is undoubtedly present. The bond lengths in κ^2^-S, N bonded ligand seems to have slightly lower values; however, the difference is within the value of the standard deviation. However, the topological analysis confirms the involvement of the N28 atom in bonding with the Sb atom, not only by finding the appropriate critical point between the Sb and N atoms but also by the changes in the characteristics of the other, related bonds. As far as the thiosemicarbazones are conjugated molecules, one has to analyze not only the close vicinity of the N28 atom, but also extend the analysis to further atoms/bonds. The representative results have been shown in Figure 11, which shows that although the differences between the bonds N28-C27 and N8-C7 are negligible, the characteristics of the CPs change for the other bonds. Consequently, the most significant discrepancies are observed for the N28-N29 bond, and some minor ones for C12-N11, N11-C10, and N9-C10.The increase in the electron density at the critical point is present for N28-N29 and N31-C30, while a decrease for N31-C32 and C30-N29. This redistribution of electron density seems to stabilize the donating behavior of the N28 and S30 atoms.

The analysis of the intermolecular interactions within all three complexes shows that the N-H···Cl/O hydrogen bonds are the most significant driving forces of the crystal architecture. Additionally, in complex **1**, besides the halogen···chalcogen contact mentioned earlier, medium strength N-H···Cl and weak C-H···Cl hydrogen bonds are observed. The bridging sulfur atoms in all the cases act as acceptors for relatively strong N-H···S hydrogen bonds, which form $R_{2}^{2}$(8) dimers. The monodentate sulfur atoms, in turn, are accepting only quite weak and not very directional C-H···S interactions. The lack of weakly bonded, bidentate S ligands in complex **2** results in not forming the N-H···S hydrogen bond dimers. Consequently, the sulfur atoms are, similarly to complex **1**, involved only in weak C-H···S interactions and halogen···chalcogen contacts; in this case, the chlorine atom from dichloromethane acts as an acceptor. In complex **3,** the presence of ethanol introduces another good acceptor of hydrogen bond (oxygen atom); consequently, N-H···Cl and N-H···O are the most significant contacts in the crystal architecture, also here quite strongly bonded to the metallic center sulfur atom do not form N-H···S hydrogen bonds. Only the most important intermolecular interactions were analyzed; however, there are also more or less common contacts, like π···π, Cl···π, and S···π, which obviously have, however minor, contributions to the formation of the crystalline structure.

### 2.3. Spectroscopy

The FT-IR spectra of the thiosemicarbazones and their metal complexes provide information about the ligand binding type to the metal ion [22,23] The most significant IR spectral bands with their tentative assignments of the complexes are reported in the experimental section (Figures S1–S3). The IR spectra of the complexes reveal the presence of ν(N-H) bands due to the –NH group in the range of 3460–3286 cm^−1^. In addition, the presence of ν(N-H) bands depending on the –NH_2_ group in the range of 3286–3171 cm^−1^ supports that the ligands are coordinated to the metal ion in neutral form. The strong absorption observed at 1583–1533 cm^−1^ in the IR spectra of the free ligands is attributed to the presence of the ν(C=N) group. In the complexes, this band is observed as a positive shift in the 1589–1556 cm^−1^ range.In the literature, it has been reported that there is a negative shift in the ν(C=N) band when the thiosemicarbazones are coordinated through the imine nitrogen atom. However, in complex **2**, although one of the two ligands is coordinated to the central atom via the imine nitrogen atom, no negative shift was observed in the ν(C=N) band. According to the structural data of complex **2**, the C=N bond length in both ligands is 1.29(1) Å. This may be the reason why no negative shift was observed for the ν(C=N) band in the IR spectrum of complex **2**. While the diagnostic ν(C=S) band is observed in the range of 842–779 cm^−1^ in the free ligands, this band is shifted to a lower frequency in the complexes (820–750 cm^−1^), indicating the coordination of sulfur. In the low-frequency region of the Raman spectra of the complexes (Figures S4–S6), some new bands have been recognized in the range of 650–100 cm^−1^ [24,25]. The new bands at 253 and 233 cm^−1^ in the Raman spectra of complexes **1** and **2** have been assigned to the ν(Sb-S) vibrations, while the band appearing at 412 (3) cm^−1^ in the Raman spectrum of complex **3** is attributed to the ν(Bi-S) vibrations. Also, a new band was observed at 630 cm^−1^ in the Raman spectrum of complex **2**, which is attributed to the ν(Sb-N) vibration. In the Raman spectra of the chloro-complexes of antimony and bismuth (**1**–**3**), the bands in the 317–250 cm^−1^ region are attributed to M-Cl bonds. The NMR spectra of complexes **1**–**3** (Figures S7–S12) have been recorded in DMSO-d_6_ and various proton and carbon signals are listed in the experimental section. In the 1H-NMR spectra of these complexes, a signal due to the NH proton was observed in the range 10.25–11.54 ppm; the presence of this signal indicates that there is no deprotonation at the proton of the azomethine group and that the ligand is coordinated to the center ion into the thione form. The ^13^C NMR spectra of the complexes **1**–**3** exhibited resonance due to thiocarbonyl (C=S) carbon in the region, 179.61, 179.56, and 178.67, respectively. This carbon signal in the complexes exhibits a slight up-field shift compared to the free ligands and this can be attributed to the coordination of the thione moiety to the center atom. The UV-Visible spectra of the complexes (Figure S13) in dimethyl sulfoxide revealed mainly one strong absorption band (313 nm (**1**), 312 nm (**2**), and 320 nm (**3**)) which was assigned to intra-ligand charge transfer (n→π*).

### 2.4. Thermal Analysis

The TG-DTA curves of antimony (III) and bismuth (III) complexes heated from ambient temperature to 1000 °C under nitrogen gas are shown in Figure S14. The decomposition pattern of the first three complexes (**1**–**3**) is similar. The complexes **1**–**3** are thermally stable up to 100 (1), 100 (2), and 60 (3) °C, respectively. The TGA curves of the complexes show that the decomposition occurs in four decomposition steps. In the first step, dichloromethane molecule is released for complexes **1** and **2** (100–160 °C, exp.; 6.1%, calc.; 6.4% (**1**); exp.;11.8%; calc.; 11.67% (**2**)) and ethanol molecule is released for complex **3** (60–110 °C, exp.; 4.4%, calc.; 4.8%).In the second weight loss step, the ligand molecules dissociate in each of the three complexes (160–400 °C, exp.; 58.64%, calc.; 58.82%, four ligand molecules (**1**); 160–290 °C, exp.; 56.69%, calc.; 56.97%, two ligand molecules(**2**); 110–400 °C, exp.; 62.14%, calc.; 61.60%, three ligand molecules (**3**)). The third decomposition step for each complex occurs at 400–625 °C (**1**), 290–557 °C (**2**), and 400–790 °C (**3**), respectively, with a mass loss of 16.28% (calc. 16.18%) (**1**), 14.76% (calc. 14.61%) (**2**), and 11.85% (calc. 11.30%) (**3**) corresponding to halogen ions. The fourth decomposition step corresponds to the mass loss up to 1000 °C after the third decomposition step for each complex, which is the antimony residue for complexes**1** and **2** and bismuth residue for complex **3**.

### 2.5. Stability of Antimony (III) and Bismuth (III) Complexes in Solution

Since the stability of Sb and Bi-thiosemicarbazone complexes is an important factor affecting biological studies, the stability of the complexes was tested by monitoring the molar conductivity values and UV-Vis spectra. No significant difference was observed in the molar conductivity values of the complexes in 10^−3^ M dimethyl sulfoxide solution for 0, 24, and 48 h (Table S2 Supplementary Materials (SM)). The stability of the complexes in dimethylsulfoxide and pH 7.4 phosphate-buffered solution was tested by UV-Vis spectroscopy over a period of 48 h and no significant change was observed in the spectral properties and peak absorption of the complexes over time (Figures S15–S16 (SM)). This confirms the stability of the complexes in organic solvent and phosphate-buffered solution. The stability of the main group metal complex solutions makes them good candidates for biological applications.

### 2.6. Biological Results

#### 2.6.1. Antiproliferative Study

The aromatic thiosemicarbazones and their antimony (III) and bismuth (III) halide complexes were tested for their cytotoxic activity against the human cervical adenocarcinoma cell line (HeLa). The IC_50_ values of complex 3 and the free ligands were higher than 30 μM against the HeLa cell line (Table 3), indicating that these complex and free ligands were not active against this cell line. On the other hand, the IC_50_ values of complexes 1 and 2 are lower than 30 μM and these complexes show a modest antiproliferative activity against the HeLa cell line. In general, the antimony (III) complexes of aromatic thiosemicarbazones showed a more selective antiproliferative activity against the HeLa cells than the bismuth (III) complexes [14,15]. However, the antiproliferative activity of the antimony (III) complexes of thioamides and dithiocarbamates against this cell line is much higher than that of the antimony (III) complexes of aromatic thiosemicarbazones [24,26,27,28,29]. When this situation was evaluated for the bismuth (III) complexes, the bismuth (III) complexes of heterocyclic thiosemicarbazones and dithiocarbamates showed a more selective antiproliferative activity against the HeLa cells than the bismuth (III) complexes of aromatic thiosemicarbazones and thioamides [30,31,32,33]. In conclusion, both the type of metal ion and the ligand significantly influence the activity of the compounds. While it is not entirely clear why the antimony compounds of thioamides exhibit greater activity than their bismuth counterparts with the same ligand, the increased toxicity of the antimony (III) ions may be related to their enhanced activity. Furthermore, among the compounds of the same metal with different ligands, dithiocarbamates complexes are more active than that of thioamides or thiosemicarbazones or thioamides, likely due to the higher stability of the former.

#### 2.6.2. Antimicrobial Study

**Figure 12:**
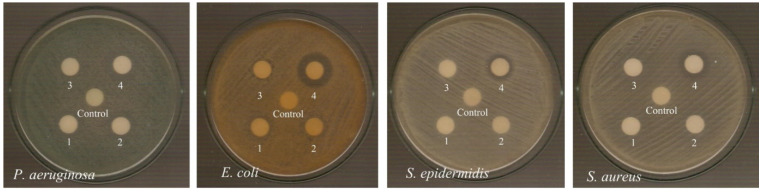
Inhibition zones which are developed in the agar plates of *P. aeruginosa, E. coli, S. epidermidis, and S. aureus* by the antimony (III) and bismuth (III) thiosemicarbazone complexes **1**–**3** at 1mM. NB: sample **4** is not connected with the present study.

All the complexes and free thiosemicarbazone ligands were assessed for their antibacterial activity against Gram-negative (*P. aeruginosa* and *E. coli*) and Gram-positive (*S. epidermidis* and *S. aureus*) bacteria. The antibacterial data are summarized in Table 4 and presented in Figure 12 which show that the antimony (III) and bismuth (III) aromatic thiosemicarbazone complexes have greater antimicrobial activity against Gram-negative bacteria compared to the free ligands. On the other hand, neither other complexes nor free ligands were effective against Gram-positive bacteria. In general, the thiosemicarbazone complexes of main group elements are much more effective against Gram-negative bacteria than Gram-positive bacteria [14,15,25,30].

## 3. Materials and Methods

### 3.1. Materials and Instruments

Acetophenone, benzaldehyde, thiosemicarbazide, 4-methyl-3-thiosemicarbazide, antimony (III) chloride, bismuth (III) chloride, and all the solvents were sourced from Merck and Sigma-Aldrich (Merck Group, Darmstadt, Germany) with high analytical gradients and used as received without purification in the experiment. All the melting points were measured on a STUART SMP30 (Stuart, Cincinnati, OH, USA) melting point apparatus and were uncorrected. The molar conductivity measurements were carried out at 25 °C in DMSO and DMF solutions with a VWR Phenomenal CO 3000 L conductivity meter (Avantor, Radnor, PA, USA). The C, H, N, and S analyses were performed via a Carlo Erba EA MODEL 1108 elemental analyzer (Waltham, MA, USA). The FT-IR spectra were gained in the range of 4000–400 cm^−1^ via a Vertex 70 IF-IR spectrometer (Brucker Optics GmbH & Co. KG, Ettlingen, Germany) using the ATR technique. The Raman spectra of the complexes were measured by a Renishaw in Via Reflex Laser Raman spectrometer (Wotton-under-Edge, Gloucestershire, UK, 785 nm excitation). The UV-Vis spectra were recorded using a UV-Vis spectrophotometer UV 2600 (Shimadzu, UV-1900, Kyoto, Japan). The1H and 13C NMR spectra were acquired using an Agilent Premium Compact 600 MHz spectrophotometer (Agilent Technologies, Santa Clara, CA, USA). The thermogravimetric analysis (TGA) experiments were carried out in the temperature range of 25–800 °C with a Hitachi EXSTAR SII 7300 (Hitachi High-Tech Science Corporation, Tokyo, Japan) instrument in a nitrogen atmosphere at a heating rate of 5 °C min^−1^.

### 3.2. Single Crystal X-ray Measurement and Refinement

The X-ray diffraction data were collected by the ω-scan technique on a four-circle Rigaku Rigaku Europe, Neu-Isenburg, GermanyXcalibur diffractometer (equipped with an Eos detector equipped with a graphite-monochromized MoKα radiation source (λ = 0.71073 Å) [35]. The data were corrected for Lorentz-polarization effects and for absorption [36]. Accurate unit cell parameters were determined by a least-squares fit of the reflections of the highest intensity, which were chosen from the whole experiment. The calculations were mainly performed within the OLEX2 v1.5 [37] and WinGX ver. 2023.1 program systems [38]. The structures were solved with ShelxT [39] and refined with the full-matrix least-squares procedure on F^2^ by SHELXL [40]. Scattering factors incorporated in SHELXL were used. All the non-hydrogen atoms were refined anisotropically, and the hydrogen atoms were located at the calculated positions and refined as a “riding model” with isotropic thermal parameters fixed at 1.2 times the U_eq_ of the appropriate carrier atom for compounds. For compound **1,** a solvent mask was calculated and used and 40 electrons were found in a volume of 119Å^3^ in **1** void per unit cell. This is consistent with the presence of 2 water molecules per asymmetric unit which account for 40 electrons per unit cell. The crystal data and refinement parameters are presented in Table 5. Topological analysis has been proceed using MoPro Suite (ver. 2023.09) software [41] and the structure visualization has been prepared with Mercury (ver. 2022.3.0) [42] and MoProViewer (ver. 2023.09) [41] software.

The crystallographic data for the structural analysis has been deposited with the Cambridge Crystallographic Data Centre, No. CCDC 2310421 **1** (**α**), 2376681 **1** (**β**), 2310419 (**2**), and 2310420 (**3**).

### 3.3. Powder Crystal X-ray Measurement

The powder X-ray studies have been performed with on Bruker D8 Quest (Billerica, 40 Manning Rd, USA) with (Cu) X-ray Source with Photon III detector (CuK_α_ radiation (λ = 1.54178 Å). The 2θ range for the measurement was 0–35° with 30 s exposure time. Diffractograms were analyzed with the KDif v.3.01b program from the Kalvados package (Prague, Czech Republic) [43].

### 3.4. Preparation of Complexes

#### 3.4.1. Preparation of {[SbCl_3_(µ_2_-S-Hacptsc)(η^1^-S-Hacptsc)], 2/3H_2_O,1/3CH_2_Cl_2_ (**1**)

In a 50 mL round-bottom flask, acetophenone thiosemicarbazone (0.193 g, 1.0 mmol) was dissolved in 10 mL of dichloromethane. A total of 0.5 mmol of antimony (III) chloride (0.114 g) was dissolved in 10 mL dichloromethane and added slowly to the thiosemicarbazone solution. The reaction was stirred at room temperature for 5 h and the solution was refrigerated for 1–2 days to obtain yellow crystals. The yield was 0.243 g (74.08%), melting point: 102–104 °C (with decomposition), and m.w.: 1314.22 g·mol^−1^. Elemental analysis: Calculated: for C_37_H_46_Cl_8_N_12_S_4_Sb_2_: C, 33.82; H, 3.53; N, 12.79; S, 9.76%. Found: C, 33.80; H, 3.54; N, 12.76; S, 9.73%. It is soluble in acetone, acetonitrile, dichloromethane, dimethyl sulfoxide, methanol, and tetrahydrofuran. Λ_M_ (DMSO): 21.60 ± 0.65 Ω^−1^cm^2^mol^−1^, Λ_M_ (DMF): 20.50 ± 1.08 Ω^−1^cm^2^mol^−1^. IR (cm^−1^): ν(N-H) 3460, 3416, ν(N-H) 3273, ν(C=N) 1589, ν(N-N) 1078, ν(C=S) 820. ^1^H NMR (400 MHz, DMSO-*d_6_*, δ, ppm): 10.28 (s, 2H, N^2^H), 8.34 (s, 4H, N^1^H), 8.01–7.97 (m, 4H, C^4^H, C^8^H), 7.45–7.36 (m, 6H, C^5^H, C^6^H, C^7^H), 2.33 (s, 6H, -CH_3_). ^13^C NMR (400 MHz, DMSO-*d*_6_, δ, ppm): 179.61 (C1), 149.14 (C2), 138.57 (C3), 130.30 (C6), 129.29 (C5–C7), 127.62 (C4–C8), 15.13 (-CH_3_).

#### 3.4.2. Preparation of {[SbCl_3_(κ^2^-S, N-Hacpmtsc)(η^1^-S-Hacpmtsc)].CH_2_Cl_2_} (**2**)

In a 50 mL round-bottom flask, acetophenone N-methyl-thiosemicarbazone (0.207 g, 1.0 mmol) was dissolved in 10 mL of methanol. To the methanol solution of thiosemicarbazone was added the dichloromethane solution (10 mL) of antimony (III) chloride (0.114 g, 0.5 mmol). The solution was stirred at room temperature for 3 h and the solution was refrigerated overnight to obtain pale yellow crystals. The yield was 0.283 g (77.82%), melting point: 136–139 °C (with decomposition), m.w.: 727.63 g mol^−1^. Anal. calc. for C_21_H_28_Cl_5_N_6_S_2_Sb: C, 34.66; H, 3.88; N, 11.55; S, 8.81%. Found: C, 34.59; H, 3.85; N, 11.56; S, 8.83%. It is soluble in acetone, acetonitrile, dichloromethane, dimethyl sulfoxide, methanol, and tetrahydrofuran. Λ_M_ (DMSO): 22.50 ± 0.85 Ω^−1^cm^2^mol^−1^, Λ_M_ (DMF): 20.60 ± 1.09 Ω^−1^cm^2^mol^−1^. IR (cm^−1^): ν(N-H) 3296, ν(N-H) 3198, ν(C=N) 1556, ν(N-N) 1030, ν(C=S) 827. ^1^H NMR (400 MHz, DMSO-*d_6_*, δ, ppm): 10.28 (s, 2H, N^2^H), 8.50 (d, *J* = 4.6 Hz, 2H, N^1^H), 7.97 (dd, *J* = 6.7, 3.1 Hz, 4H, C^4^H, C^8^H), 7.49–7.40 (m, 6H, C^5^H, C^6^H, C^7^H), 3.08 (d, *J* = 4.5 Hz, 6H, N-CH_3_), 2.33 (s, 6H, C-CH_3_).^13^C NMR (400 MHz, DMSO-*d*_6_, δ, ppm): 179.56 (C1), 148.58 (C2), 138.64 (C3), 130.16 (C6), 129.23 (C5, C7), 127.55 (C4, C8), 32.14 (C-CH_3_), 15.04 (N-CH_3_).

#### 3.4.3. Preparation of {[BiCl_3_(η^1^-S-Hbzmtsc)_3_]·C_2_H_5_OH} (**3**)

A total of 0.158 g of bismuth (III) chloride (0.5 mmol) was weighed into a 50 mL round-bottom flask and 10 mL of ethanol was added. A few drops of concentrated HCl were added to the solution and stirred until the bismuth (III) chloride was completely dissolved. A solution of benzaldehyde N-methyl-thiosemicarbazone (0.290 g, 1.5 mmol) in ethanol (10 mL) was added to the stirred solution of bismuth (III) chloride. The stirred reaction was heated at 60–65 °C for 3 h. After cooling to room temperature, a yellow precipitation was filtered off and was recrystallized from hot ethanol. For the yellow crystals, the yield was 0.365 g (77.40%), melting point: 174–176 °C, and m.w.: 941.20 g mol^−1^. Anal. calc. for C_29_H_39_Cl_3_N_9_OS_3_Bi: C, 37.01; H, 4.18; N, 13.39; S, 10.22%. Found: C, 37.07; H, 4.12; N, 13.41; S, 10.19%. It is soluble in acetone, acetonitrile, benzene, chloroform, dichloromethane, dimethyl sulfoxide, ethanol, methanol, tetrahydrofuran, and toluene. Λ_M_ (DMSO): 5.80 ± 0.25 Ω^−1^cm^2^mol^−1^, Λ_M_ (DMF): 7.96 ± 0.42 Ω^−1^cm^2^mol^−1^ IR (cm^−1^): ν(N-H) 3304, ν(N-H) 3171, ν(C=N) 1574, ν(N-N) 1088, ν(C=S) 750. ^1^H NMR (400 MHz, DMSO-d6, δ, ppm): 11.54 (d, *J* = 4.5 Hz, 3H, N^2^H),8.56 (t, *J* = 5.0 Hz, 3H, N^1^H), 8.08 (d, *J* = 4.6 Hz, 3H, C^2^H),7.88–7.80 (m, 6H, C^4^H, C^8^H), 7.51–7.41 (m, 9H, C^5^H, C^6^H, C^7^H),3.40 (d, *J* = 5.3 Hz, 9H, -CH_3_).^13^C NMR (400 MHz, DMSO-*d*_6_, δ, ppm): 178.67 (C1), 142.64 (C2), 135.23 (C3), 130.70 (C6), 129.61 (C5, C7), 128.15 (C4, C8), 31.82 (-CH_3_).

### 3.5. Antiproliferative Study

The procedure was performed as previously reported [44]. The cell viability assessment via the SRB assay was conducted using DMSO/DMEM solutions with DMSO concentrations ranging from 0.01 to 0.3% *v*/*v* in DMEM. Fresh stock solutions of these agents (0.01 M in DMSO) were prepared and diluted with cell culture medium to the desired concentration. The freshly prepared solutions were then diluted with cell culture medium to the final concentrations of 0.01–100 μM. For the experiments, the cells were plated (100 μL per well) in 96-well flat-bottom microplates at different inoculation densities (HeLa: 5000 cells/well). The cells were incubated for 24 h at 37 °C to resume exponential growth, and then exposed to the test compounds for 48 h by adding an equal volume (100 μL) of either complete culture medium (control wells) or the medium containing twice the final drug concentration (test wells). Cytotoxicity was assessed using the SRB colorimetric assay, measuring the percentage of surviving cells relative to the control (untreated cells). After the culture medium was aspirated, 50 μL of 10% cold trichloroacetic acid (TCA) was gently added to each well. The plates were incubated for 30 min at 4 °C, washed five times with deionized water, and dried at room temperature for at least 24 h. Subsequently, 70 μL of 0.4% (*w*/*v*) sulforhodamine B (Sigma) in 1% acetic acid was added to each well and incubated for 20 min at room temperature. Excess SRB was removed, and the plates were washed five times with 1% acetic acid, and then air-dried. Bound SRB was solubilized with 200 μL of 10 mM unbuffered Tris-base solution, and absorbance was read at 540 nm using a 96-well plate reader. The results were expressed as IC50 values, representing the concentration of drug required to inhibit cell growth by 50% after 48 h of incubation. For each compound tested, a dose–effect curve was generated. Sextuplicate determinations yielded a coefficient of variation (CV) between 1 and 5% with a very low standard error (SE). The cellular growth inhibition data were expressed as the fraction of unaffected cells (fu; survival fraction, SF) calculated using the equation: fu = ODx/ODc, where ODx is the optical density of the test sample and ODc is the control optical density. Drug potency was expressed as the IC50 values calculated from the dose–effect curves using least-squares regression analysis. Statistical significance was determined when *p* ≤ 0.05 (Student’s *t*-test).

### 3.6. Determination of the Inhibition Zone (IZ) through Agar Disk-Diffusion Method

The procedure was performed as previously reported [45]. For the antibacterial experiments, we utilized the strains of *Staphylococcus epidermidis* (ATCC^®^ 14,990™), *S. aureus subsp. aureus* (ATCC^®^ 25,923™), *Pseudomonas aeruginosa*, and *Escherichia coli*. The antibacterial experiments were performed in DMSO/Broth solutions, with 0–0.1% *v*/*v* DMSO in broth for the compounds. To evaluate the inhibition zones (IZs) caused by the agents, the agar disk diffusion method was employed. Agar plates were inoculated with a standardized inoculum (10^8^ cfu mL⁻^1^) of the test microorganism. Filter paper disks, approximately 10 mm in diameter, soaked in the agent’s solution (1 mM), were placed on the agar surface. The Petri dishes were incubated for 20 h, after which the diameters of the inhibition zones were measured (Figure 12, Table 4). The inhibition zones displayed in Figure 12 are not clearly visible, indeed. However, we currently do not have any more convincing techniques, such as resazurin assays [46], for the studies of antimicrobial activity.

## 4. Conclusions

Three unique, biologically active compounds with intriguing structural features have been synthesized and characterized. Due to their unusual bonding modes and geometries, a detailed topological analysis of the electron density distributions in the crystals was conducted using the Atoms-In-Molecules (AIM) approach. This analysis provided a deeper understanding of the coordination spheres of the Sb/Bi complexes. Typically, aromatic thiosemicarbazones coordinate monodentately with main group elements via the sulfur donor atom. In complex **2**, one of the two thiosemicarbazone ligands coordinates in this conventional mode, while the second ligand coordinates bidentately through N and S-donor atoms. While the S-donation is significantly stronger, the topological analysis clearly confirms the presence of the N-donation as well. To the best of our knowledge, this is the first reported instance where two binding modes of the same ligand are observed in a single crystal structure of antimony (III) halide complexes. Additionally, in complex **1**, a solid-to-solid phase transition was detected and analyzed in detail. This phase change results in the formation of a binuclear complex with a very short Sb···Sb contact supported by halogen···chalcogen interactions. The complexes were also evaluated for their biological activity, revealing higher antiproliferative and antibacterial activities than the free ligand, making them promising candidates for further studies.

## Figures and Tables

**Table 1:** Selected bond critical point characteristics.

Atom1	Atom2	Cp no	*G_cp_*	*V_cp_*	R_1,2_	*ρ_cp_*	∇^2^*ρ_cp_* I	*H_cp_*	*G_cp_*/*ρ_cp_*	*V_cp_*/*G_cp_*	E_int_
dimerA											
Sb2	S50	cp4	86.95	−126.07	2.64	0.35	1.756	−39.12	0.094	−1.45	−63.035
Sb2	S70	cp5	60.66	−83.07	2.81	0.27	1.404	−22.41	0.086	−1.37	−41.535
Sb2	S70 *	cp6	18.65	−19.26	3.45	0.10	0.662	−0.61	0.072	−1.03	−9.630
dimer B											
Sb1	S10	cp13	98.70	−145.12	2.58	0.39	1.92	−46.42	0.098	−1.47	−72.560
Sb3	S90	cp14	96.21	−141.07	2.59	0.38	1.89	−44.86	0.097	−1.47	−70.535
Sb1	S110	cp15	42.50	−53.85	3.00	0.20	1.14	−11.35	0.080	−1.27	−26.925
Sb1	S30	cp16	30.05	−34.87	3.19	0.15	0.93	−4.82	0.076	−1.16	−17.435
Sb3	S30	cp17	46.91	−60.78	2.95	0.22	1.21	−13.87	0.082	−1.30	−30.390
Sb3	S110	cp18	25.70	−28.59	3.28	0.13	0.84	−2.89	0.075	−1.11	−14.295
dimer C											
Sb1	S10	cp22	92.58	−135.22	2.61	0.37	1.83	−42.64	0.10	−1.46	−67.61
Sb2	S30	cp23	25.67	−28.65	3.28	0.13	0.83	−2.98	0.07	−1.12	−14.33
Sb3	S30 *	cp24	47.87	−62.34	2.94	0.22	1.23	−14.47	0.08	−1.30	−31.17
dimer A											
Sb2	Cl5	cp1	173.42	−245.87	2.38	0.53	3.71	−72.45	0.13	−1.42	−122.94
Sb2	Cl6	cp2	98.91	−137.14	2.60	0.37	2.23	−38.23	0.10	−1.39	−68.57
Sb2	Cl4	cp3	91.70	−126.18	2.63	0.35	2.10	−34.48	0.11	−1.38	−63.09
dimer B											
Sb1	Cl2	cp7	153.58	−217.58	2.42	0.49	3.29	−64.00	0.121	−1.42	−108.79
Sb1	Cl3	cp8	95.64	−132.20	2.61	0.36	2.17	−36.56	0.102	−1.38	−66.10
Sb1	Cl1	cp9	95.05	−131.28	2.62	0.36	2.16	−36.23	0.102	−1.38	−65.64
Sb3	Cl8	cp10	160.17	−227.01	2.41	0.50	3.43	−66.84	0.122	−1.42	−113.51
Sb3	Cl9	cp11	92.12	−126.80	2.63	0.35	2.11	−34.68	0.101	−1.38	−63.40
Sb3	Cl7	cp12	98.93	−137.18	2.60	0.37	2.23	−38.25	0.103	−1.39	−68.59
dimer C											
Sb1	Cl1	cp 19	96.68	−133.76	2.61	0.36	2.19	−37.08	0.10	−1.38	−66.88
Sb1	Cl2	cp20	165.18	−234.16	2.40	0.51	3.53	−68.98	0.12	−1.42	−117.08
Sb1	Cl3	cp21	99.63	−138.25	2.59	0.37	2.24	−38.62	0.10	−1.39	−69.13

* 1 − x, 1 − y, 1 − z, *G_cp_*, *V_cp_*, E_int_, *H_cp_* [kJ/mol/bohr^3^], R_1,2_—distance between atom 1 and 2 [Å], *ρ_cp_* [e/Å^3^], ∇^2^*ρ_cp_* [e/Å^5^].

**Table 2:** Selected non-covalent bond critical point characteristics.

Atom1	Atom2	Cp no	*G_cp_*	*V_cp_*	R_1,2_	*ρ_cp_*	∇^2^*ρ_cp_*	*H_cp_*	*G_cp_*/*ρ_cp_*	E_int_	Symm
Sb1	Sb3	cp101	12.21	−12.40	3.92	0.075	0.44	−0.19	0.062	−6.20	x, y, z
Cl9	S110	cp102	22.44	−20.14	3.29	0.093	0.91	2.30	0.092	−10.07	x, y, z

*G_cp_*, *V_cp_*, E_int_, *H_cp_* [kJ/mol/bohr^3^], R_1,2_—distance between atom 1 and 2 [Å], *ρ_cp_* [e/Å^3^], ∇^2^*ρ_cp_* [e/Å^5^].

**Table 3:** The IC_50_ values (μM) of the antimony (III) and bismuth (III) complexes and their ligands againstthe human cervical adenocarcinoma cell line (HeLa) after an incubation period of 48 h.

Compounds	IC_50_ (μΜ)	Ref.
**Hacptsc**	>30	*
**Hacpmtsc**	>30	*
**Hbzmtsc**	>30	*
**Complex 1**	20.6 ± 1.8	*
**Complex 2**	27.7 ± 3.4	*
**Complex 3**	>30	*
**[SbCl_3_(DMTU)_3_]**	9.0 ± 0.3	[29]
**[SbBr_3_(TMTU)_3_]**	12.7 ± 2.5	[27]
**[SbI_2_(µ_2_-I)(EtMBZIM)_2_]_2_.3H_2_O**	3.2 ± 1.7	[28]
**[SbCl_3_(µ_2_-S)(MtMBZIM)_2_]_2_**	4.72 ± 1.34	[24]
**{[SbBr(Me_2_DTC)_2_]_n_}**	0.046 ± 0.004	[26]
**([BiCl_3_(MBZT)_2_].H_2_O)**	>30	[32]
**[BiI_3_(tHPMT)_3_]**	>30	[33]
**[BiCl_3_(Hattsc)_3_]**	4.5 ± 0.4	[30]
**{[BiI_2_(µ_2_-I)(Hattsc)_2_]_2_}**	5.6 ± 0.3	[30]
**{[BiI(µ_2_-I)(Et_2_DTC)_2_]_2_}_n_**	0.05 ± 0.006	[31]

* in this work, DMTU: N,N-dimethylthiourea, TMTU: tetramethylthiourea, EtMBZIM: 5-ethoxy-2-mercaptobenzimidazole, MtMBZIM: 5-methoxy-2-mercaptobenzimidazole, Me_2_DTCH: dimethyldithiocarbamate, Et_2_DTCH: diethyldithiocarbamate, MBZT: 2-mercaptobenzothiazole, tHPMT: 2-mercapto-3,4,5,6-tetrahydropyrimidine, and Hattsc: 2-Acetylthiophene thiosemicarbazone.

**Table 4:** Inhibition zones (mm) of the antimony (III) and bismuth (III) complexes and their ligands at 1 mM against *P. aeruginosa*, *E. coli*, *S. epidermidis*, and *S. aureus* after an incubation period of 24 h.

	Inhibition Zone (mm)				Ref.
	*P. aeruginosa*	*E. coli*	*S. epidermidis*	*S. aureus*	
**DMSO**	9.0 ± 0.0	9.0 ± 0.0	9.0 ± 0.0	9.0 ± 0.0	[15]
**Hacptsc**	9.0 ± 0.0	9.3 ± 0.7	9.0 ± 0.0	9.0 ± 0.0	[15]
**Hacpmtsc**	9.0 ± 0.0	9.0 ± 0.0	9.0 ± 0.0	9.0 ± 0.0	[15]
**Hbzmtsc**	9.0 ± 0.0	9.2 ± 0.3	9.0 ± 0.0	9.0 ± 0.0	[14]
**1**	12.0 ± 0.0	11.7 ± 0.7	9.0 ± 0.0	9.0 ± 0.0	*
**2**	13.3 ± 0.5	10.8 ± 1.5	9.0 ± 0.0	9.0 ± 0.0	*
**3**	12.5 ± 1.0	10.7 ± 1.3	9.0 ± 0.0	9.0 ± 0.0	*
**{[BiCl_3_(Htetsc)_3_].CH_3_OH}**	15.0 ± 1.1	15.5 ± 1.0	14.0 ± 0.0	15.0 ± 0.0	[34]
**{BiBr_3_(Hbztsc)_3_}**	10.7 ± 0.9	12.8 ± 1.2	9.0 ± 0.0	9.6 ± 0.9	[15]
**{BiBr_3_(Hbzmtsc)_3_}**	11.0 ± 1.3	12.7 ± 1.4	9.0 ± 0.0	10.1 ± 1.1	[14]

* in this work, Htetsc: thiophene-2-carbaldehyde-N-ethyl thiosemicarbazone and Hbztsc: benzaldehyde thiosemicarbazone.

**Table 5:** Crystal data and refinement details.

	1 (α)	1 (β)	2	3
Chemical formula	C_54_H_66_Cl_9_N_18_S_6_Sb_3_ ·CH_2_Cl_2_·2H_2_0	C_54_H_66_Cl_9_N_18_S_6_Sb_3_ ·CH_2_Cl_2_·2H_2_0	C_20_H_26_Cl_3_N_6_S_2_Sb·CH_2_Cl_2_	C_27_H_33_BiCl_3_N_9_S_3_· C_2_H_6_O
Formula weight	1314.22	1314.22	727.63	941.20
Crystal system,	Triclinic	Triclinic	Triclinic	Triclinic
Space group				
Temperature (K)	100.00(10)	293(2)	138.00(50)	100.00(10)
*a*, *b*, *c* (Å)	14.1625(5), 16.2995(7), 18.7401(7)	8.4190(2), 10.9693(3), 14.5152 (4)	9.1243(3), 9.6716(4), 10.1365(3)	9.1909(5), 9.3371(5), 12.2582(5)
α, β, γ (°)	66.292(4), 82.772(3), 75.475(3)	84.163(2), 87.856(2), 83.201 (2)	98.337(3), 105.052(3), 117.973(4)	107.110(4), 94.469(4), 108.663(5)
*V* (Å^3^)	3832.8(3)	1323.72 (6)	724.61(5)	935.17(9)
*Z*	2	2	1	1
μ (mm^−1^)	1.65	1.53	13.33	5.13
No. of measured, independent and observed [*I* > 2s(*I*)] reflections	29377, 14804, 12546	29518, 5645, 5061	11970, 5496, 5475	12760, 7454, 7449
*R*_int_	0.0220	0.040	0.0463	0.0238
*R*[*F*^2^> 2s(*F*^2^)], *wR*(*F*^2^), *S*	0.030, 0.072, 1.09	0.031, 0.079, 1.07	0.038, 0.104, 1.05	0.025, 0.058, 1.05
No. of reflections	14804	5645	5496	7454
No. of parameters	844	273	321	421
Δρ_max_, Δρ_min_ (e·Å^−3^)	1.653–0.956	0.59−0.85	1.203–1.039	1.445–1.168
CCDC number	2310421	2376681	2310419	2310420

## Data Availability

Crystallographic data for the structural analysis has been deposited with the Cambridge Crystallographic Data Centre, Nos. CCDC—2310421 (**1**), 2310419 (**2**), 2310420 (**3**), and 2338577 (**4**). Copies of this information may be obtained free of charge from The Director, CCDC, 12 Union Road, Cambridge, CB2 1EZ, UK. Fax: +44-(1223)-336-033, e-mail: deposit@ccdc.cam.ac.uk, or www: www.ccdc.cam.ac.uk.

## Supplementary Materials

The following supporting information can be downloaded at https://www.mdpi.com/article/10.3390/ijms251910794/s1.
