# Supplementary material for: Group VA Aromatic Thiosemicarbazone Complexes: Synthesis, Characterization, Biological Activity, and Topological Studies
Source: Int J Mol Sci. 2024 Oct 8;25(19):10794. doi: 10.3390/ijms251910794 (PMC11476362; doi:10.3390/ijms251910794)
Supplement: Supplementary file 1 [file ijms-25-10794-s001.zip › ijms-3226028-supplementary.pdf]

Main-group metal complexes of aromatic thiosemicarbazones: synthesis, characterization, biological activity and topological studies

Ibrahim I. Ozturk[a, b*] Emine I. Sumer[a], Anita M. Grześkiewicz[c], MaciejKubicki[c], Christina N. Banti[d], Sotiris K. Hadjikakou[d,e],

[a]*Section of Inorganic Chemistry, Department of Chemistry, TekirdagNamık Kemal University, 59030, Tekirdag, Turkiye*

[b]*Ethnochem Limited Company, Silahtaraga,University 1st Street, 13/1, Z102,Tekirdağ, Turkiye*

[c]*Department of Chemistry, A.Mickiewicz University, ul. UniwersytetuPoznanskiego 8, Poznan 61-614, Poland*

[d]*Department of Chemistry, University of Ioannina, Biological Inorganic Chemistry Lab, 45110 Ioannina, Greece*

[e]*University Research Center of Ioannina (URCI), Institute of Materials Science and Computing, Ioannina, Greece*

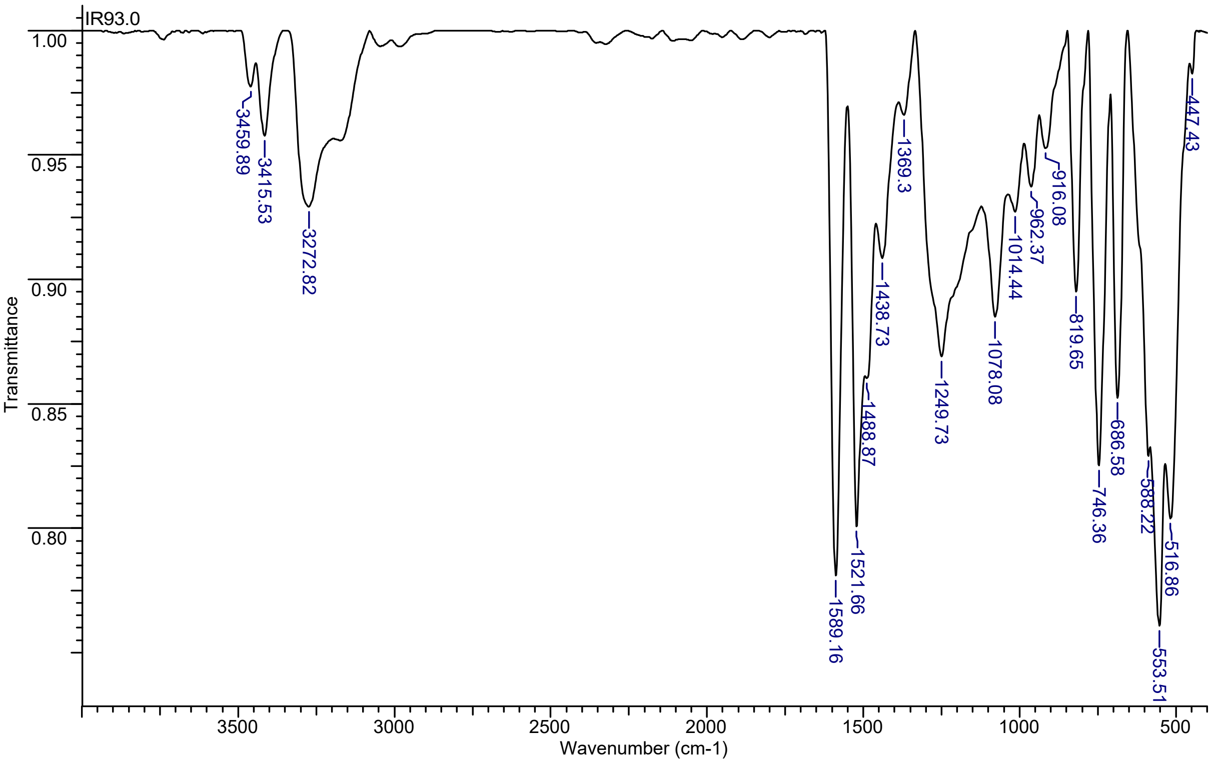

Figure S1. FT-IR spectrum of {[$SbCl_3$(μ_2-S-Hacptsc)(η^1-S-Hacptsc)]$_2$·CH_2Cl_2} (**1**).

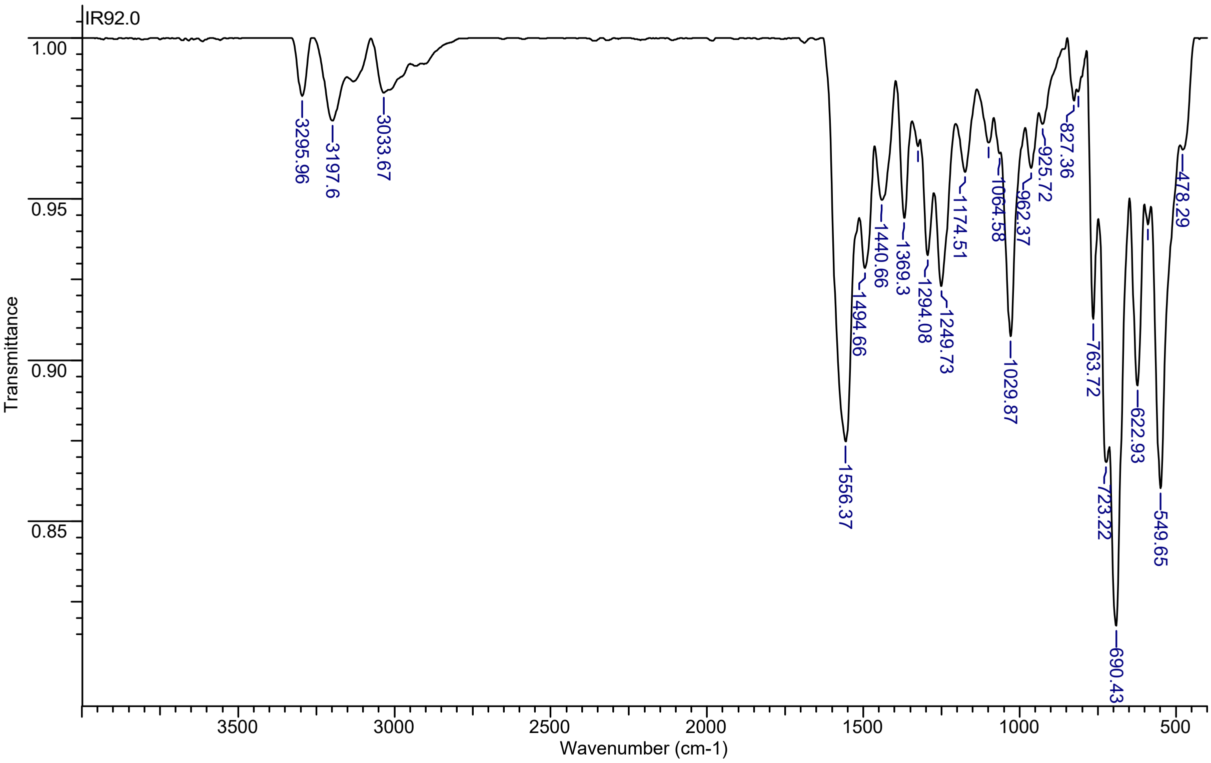

Figure S2. FT-IR spectrum of {[$SbCl_3$(κ^2-S,N-Hacpmtsc)(η^1-S-Hacpmtsc)$_2$]·CH_2Cl_2} (**2**).

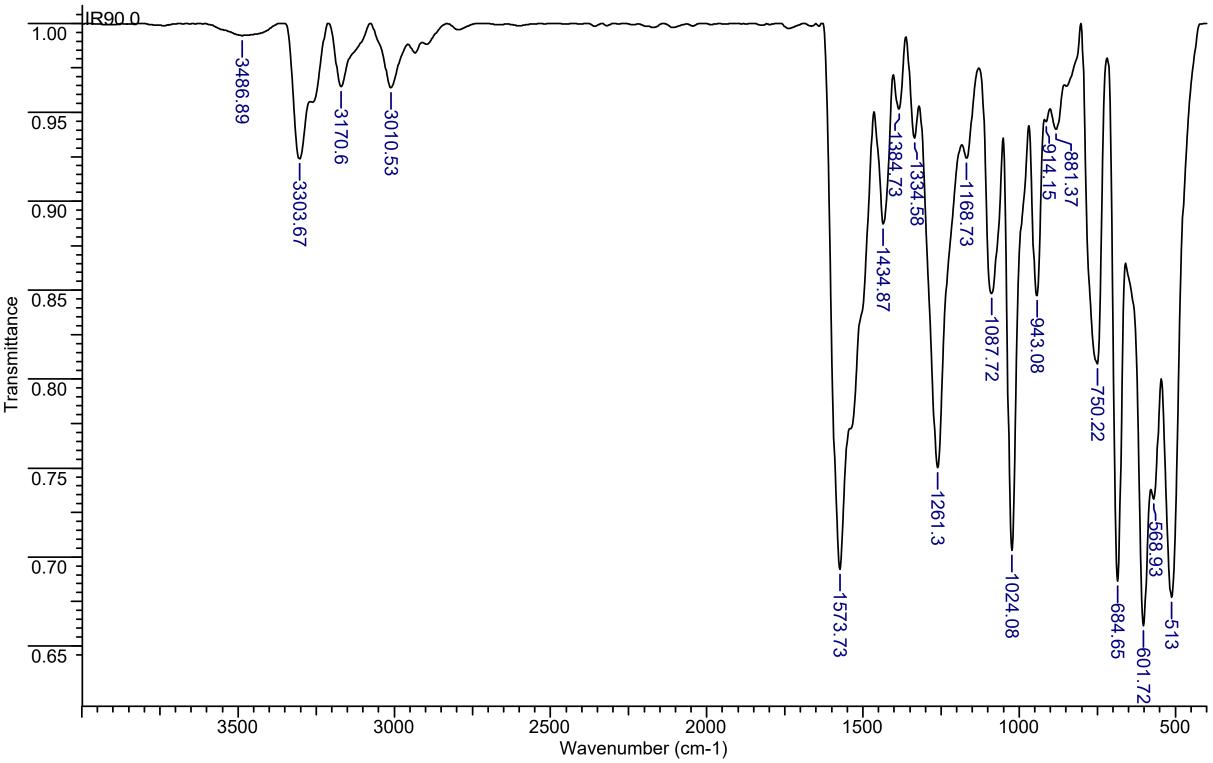

Figure S3. FT-IR spectrum of $\{[BiCl_3(\eta^1\text{-S-Hbzmtsc})_3]\cdot C_2H_5OH\}$ (**3**).

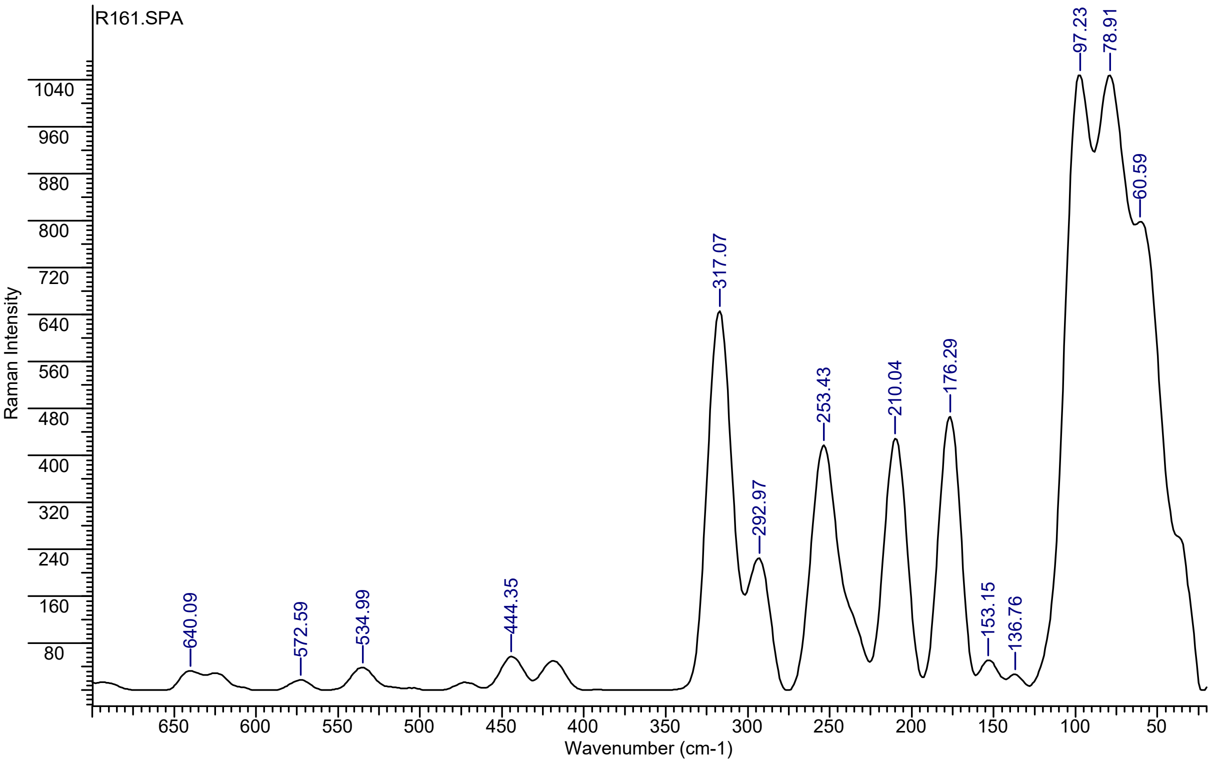

Figure S4. Raman spectrum of $\{[SbCl_3(\mu_2\text{-S-Hacptsc})(\eta^1\text{-S-Hacptsc})]_2\cdot CH_2Cl_2\}$ (**1**).

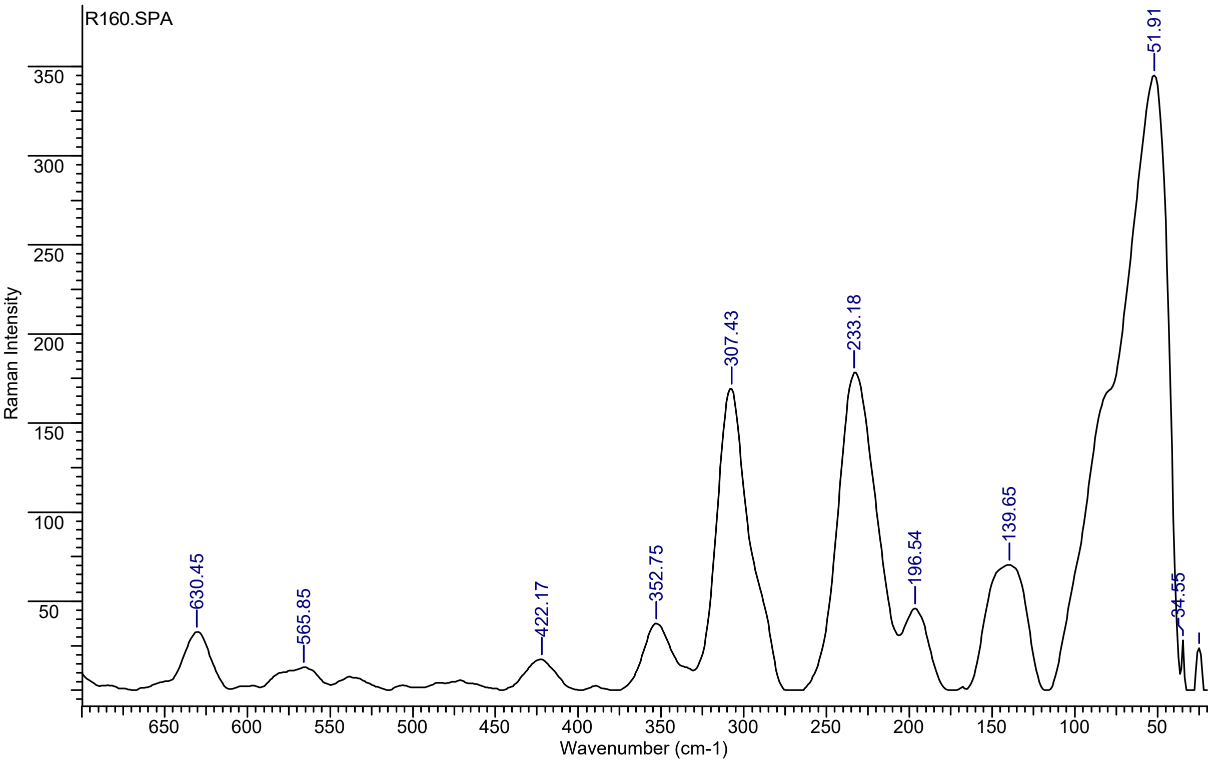

Figure S5. Raman spectrum of {[$SbCl_3$(κ^2-S,N-Hacpmtsc)(η^1-S-Hacpmtsc)$_2$]·CH_2Cl_2} (**2**).

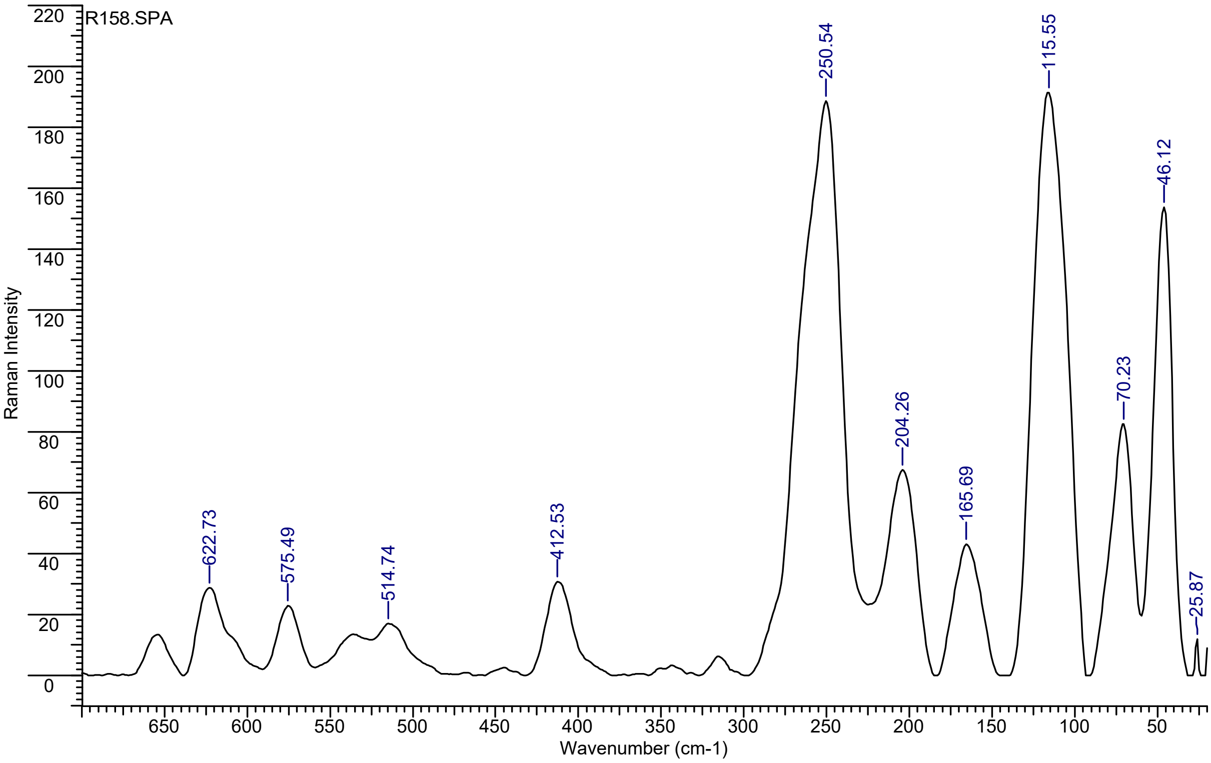

Figure S6. Raman spectrum of {[$BiCl_3$(η^1-S-Hbzmtsc)$_3$]·C_2H_5OH} (**3**).

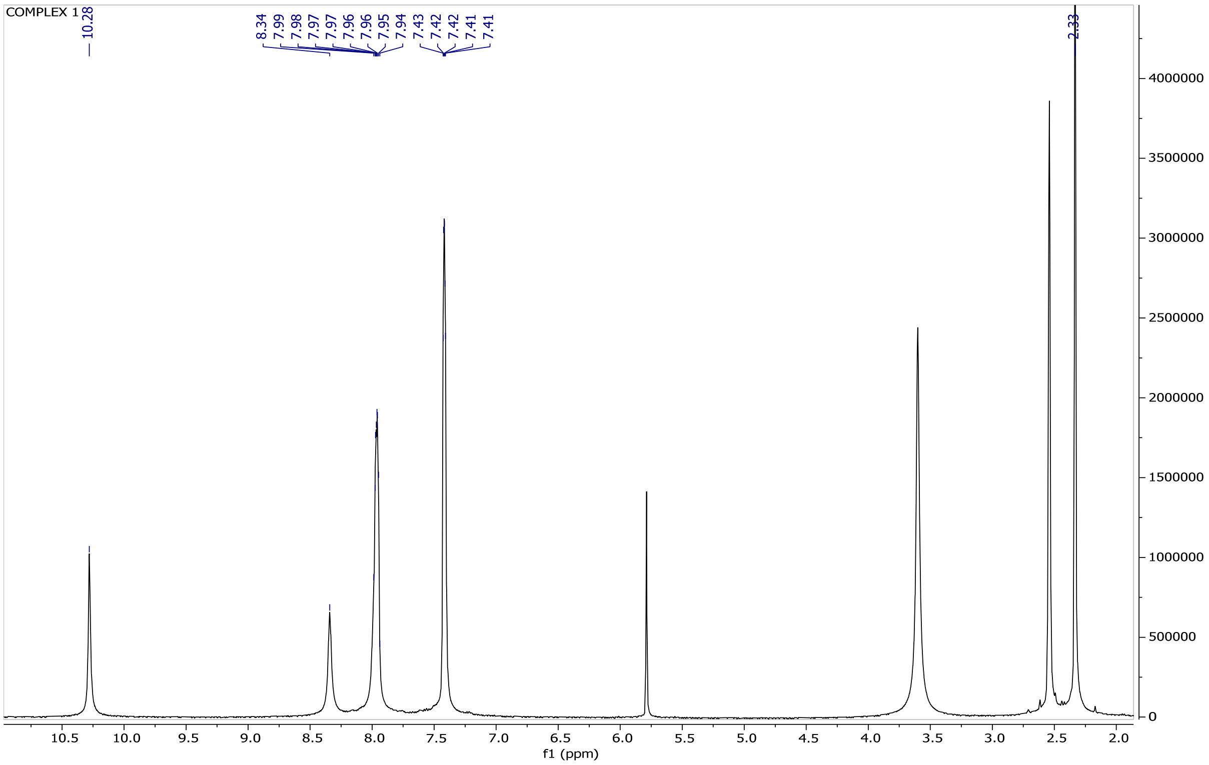

Figure S7. ^{1}H-NMR spectrum of {[SbCl$_3$(μ$_2$-S-Hacptsc)(η^1-S-Hacptsc)]$_2$·CH$_2$Cl$_2$} (**1**).

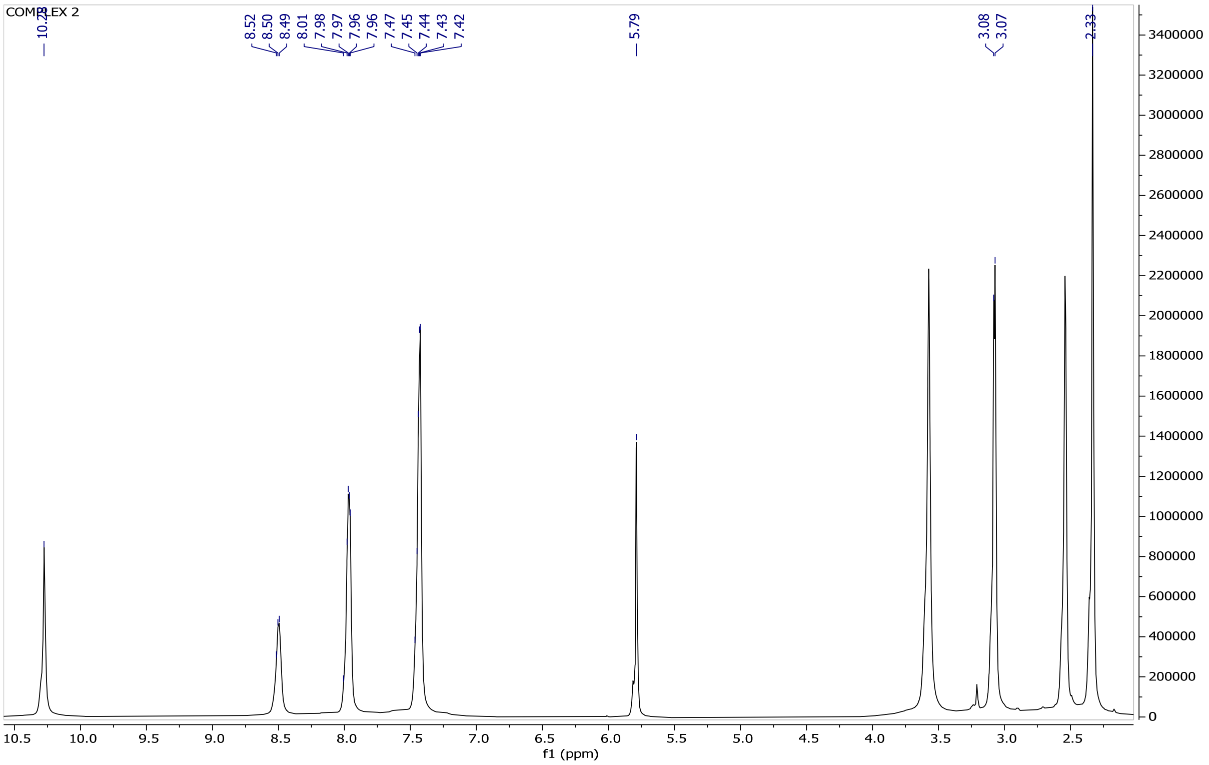

Figure S8. ^{1}H-NMR spectrum of {[SbCl$_3$(κ^2-S,N-Hacpmtsc)(η^1-S-Hacpmtsc)$_2$]·CH$_2$Cl$_2$} (**2**).

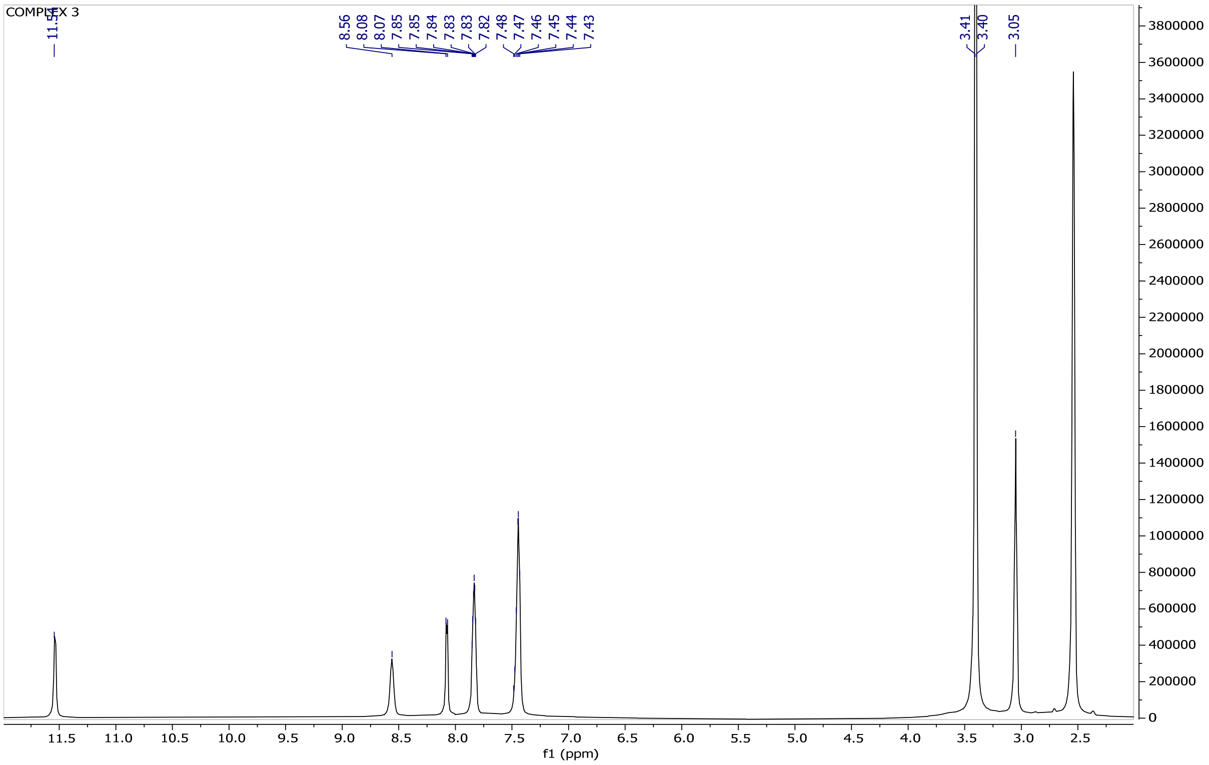

Figure S9. ^{1}H-NMR spectrum of {[BiCl$_3$(η^1-S-Hbzmtsc)$_3$]·C_2H_5OH} (**3**).

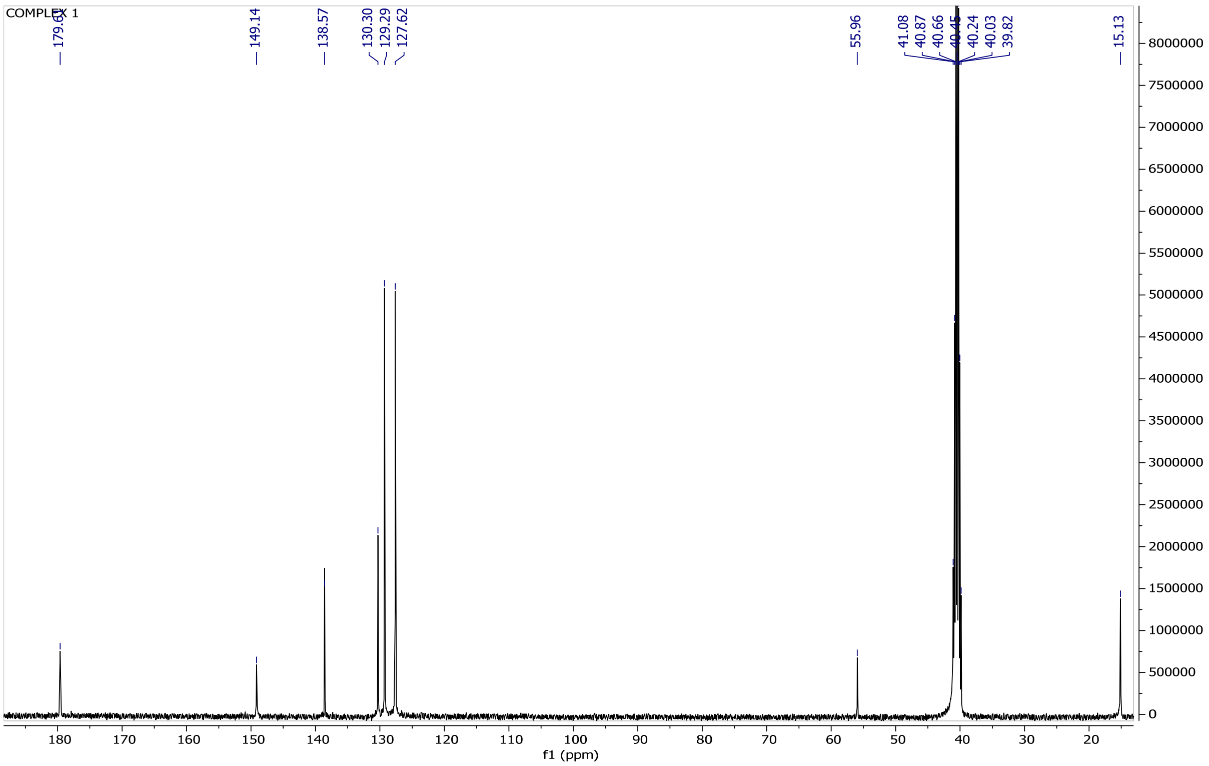

Figure S10. ^{13}C-NMR spectrum of {[SbCl$_3$(μ_2-S-Hacptsc)(η^1-S-Hacptsc)]$_2$·CH_2Cl_2} (**1**).

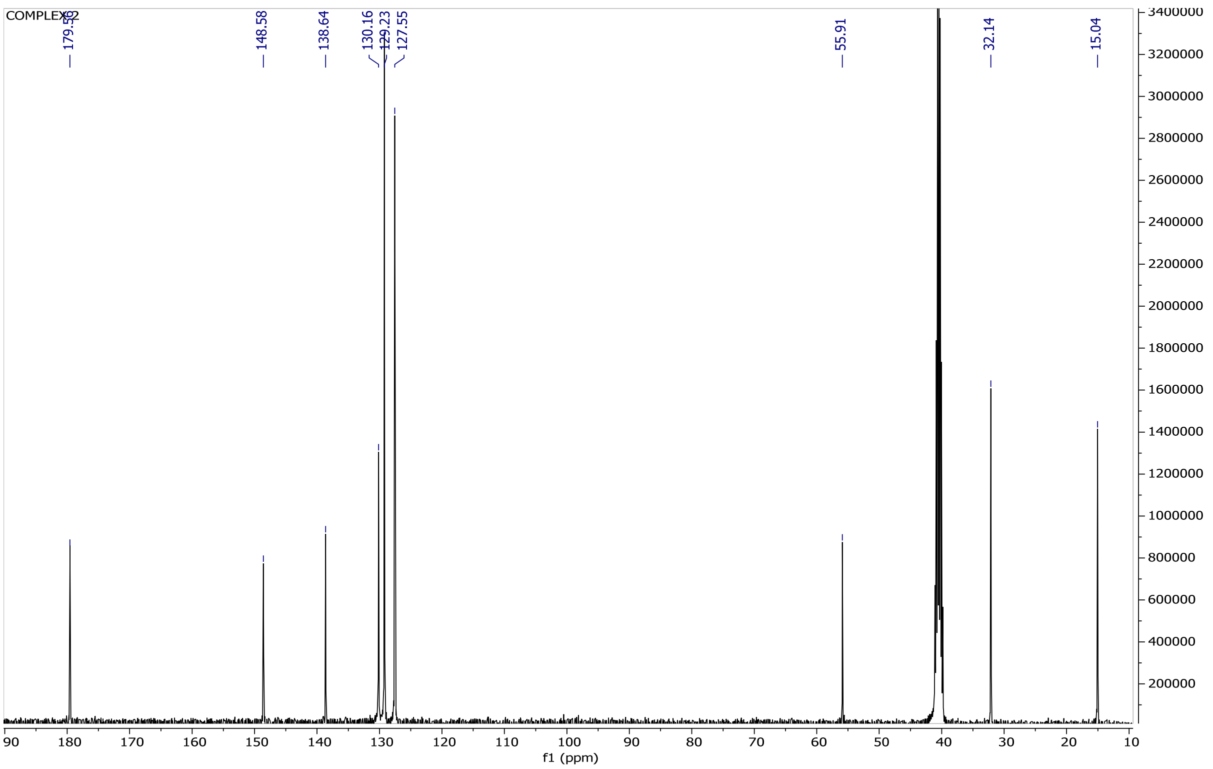

Figure S11. ^{13}C-NMR spectrum of {[SbCl$_3$(κ^2-S,N-Hacpmtsc)(η^1-S-Hacpmtsc)$_2$]$\cdot$CH$_2$Cl$_2$} (**2**).

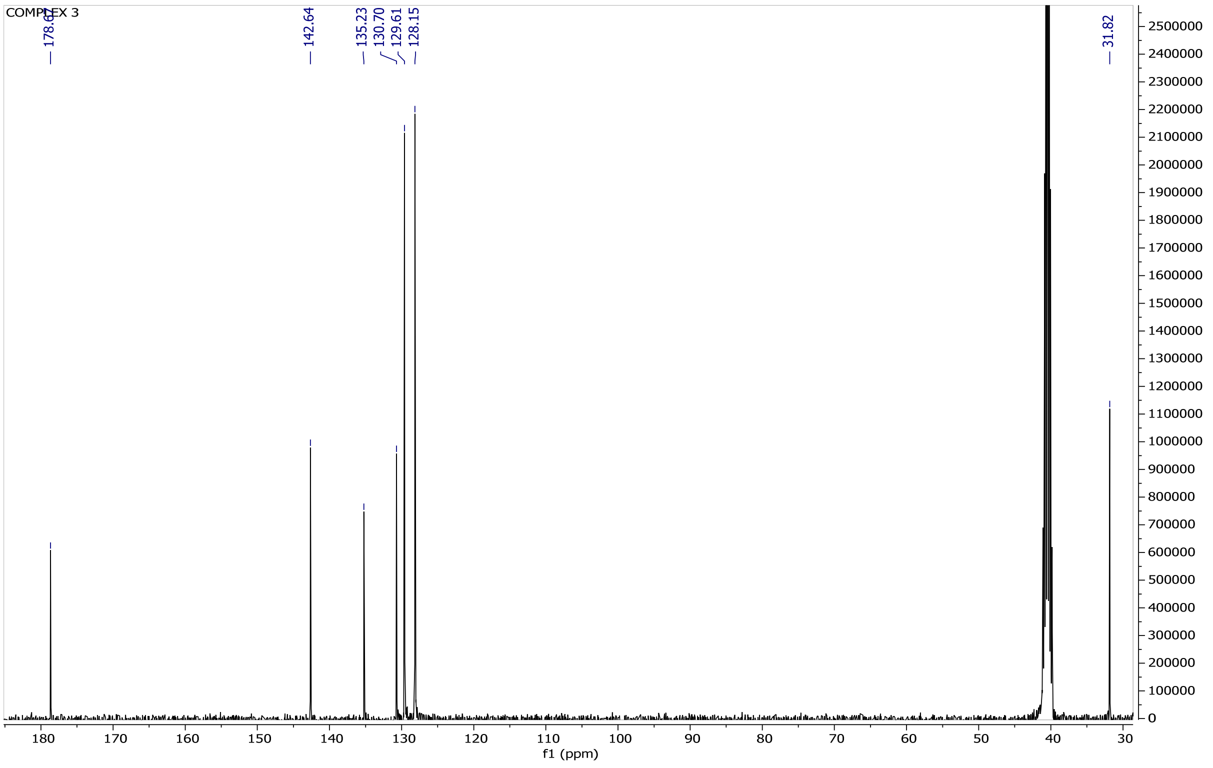

Figure S12. ^{13}C-NMR spectrum of {[BiCl$_3$(η^1-S-Hbzmtsc)$_3$]$\cdot$C$_2$H$_5$OH} (**3**).

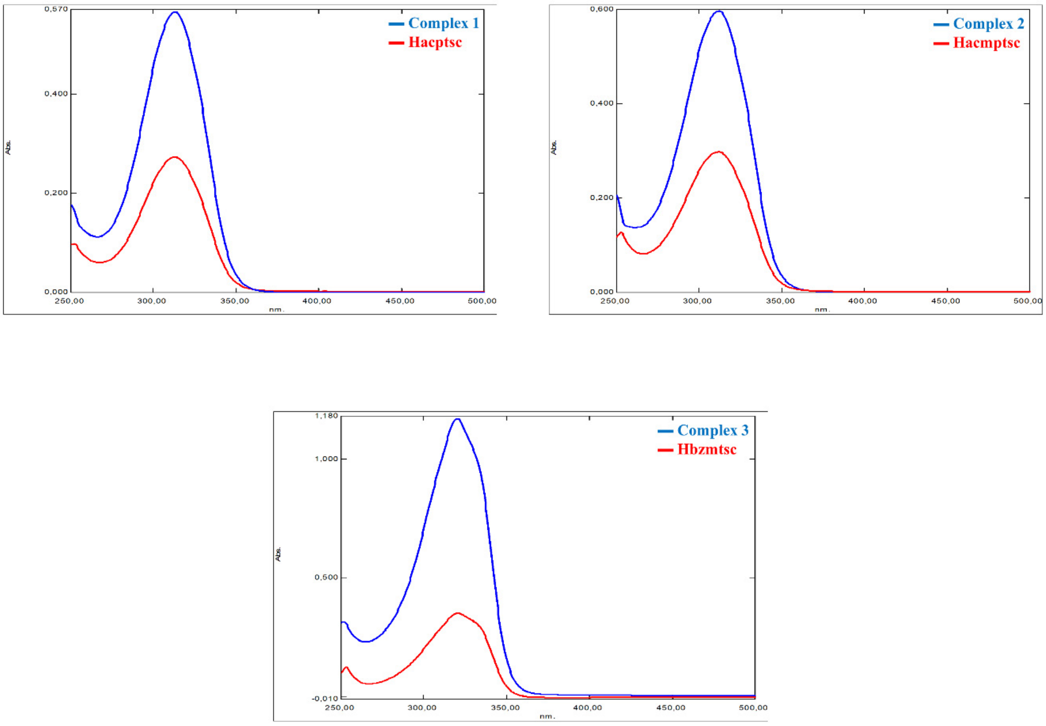

Figure s13 UV-Vis absorption spectra of the complexes (1-3) and free ligands. Spectra were recorded at room temperature and in DMSO solution (10μM).

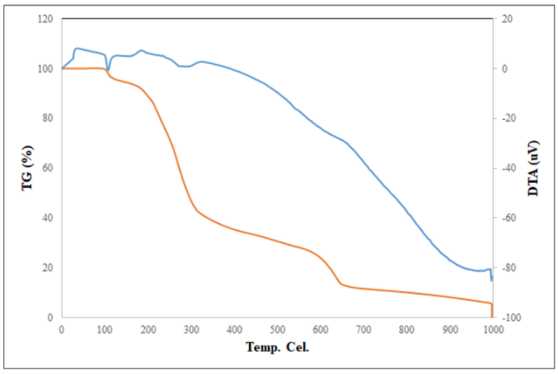

Complex 1

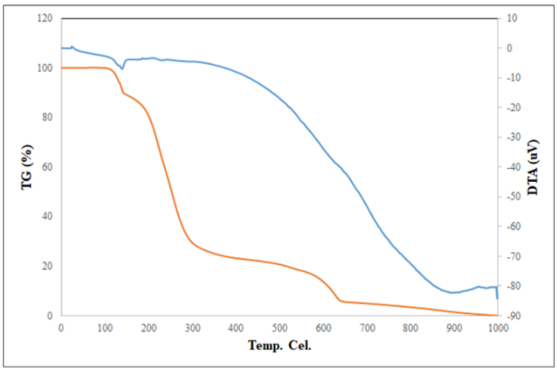

Complex 2

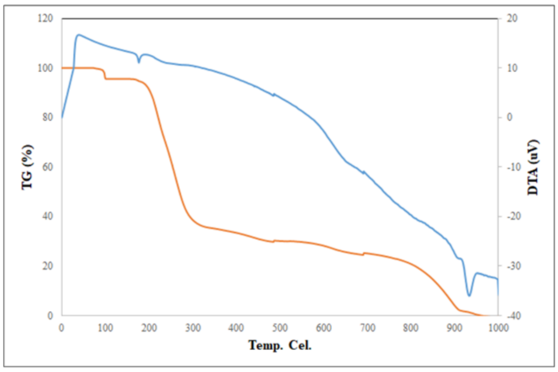

Complex 3

Figure S14. TG-DTA curves of the complexes (**1**-**3**).

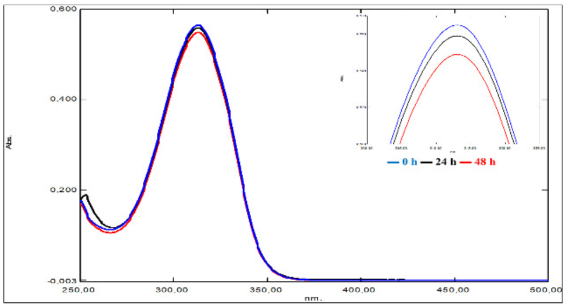

Complex 1

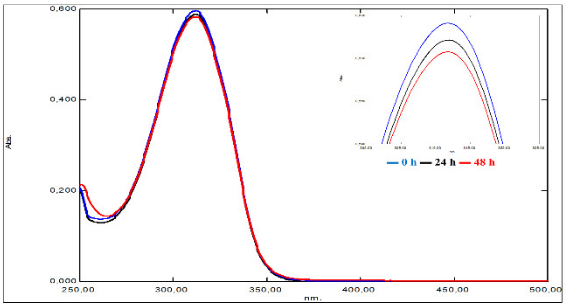

Complex 2

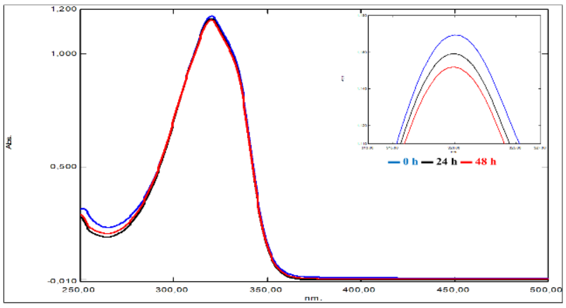

Complex 3

Figure S15. UV-Vis spectra of the complexes (**1-3**) for 0 h, 24 h and 48 h. Spectra were recorded at room temperature and in DMSO solution (10μM).

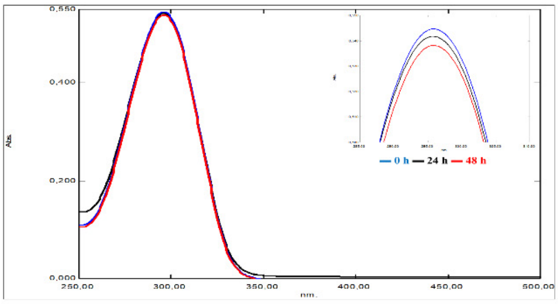

Complex 1

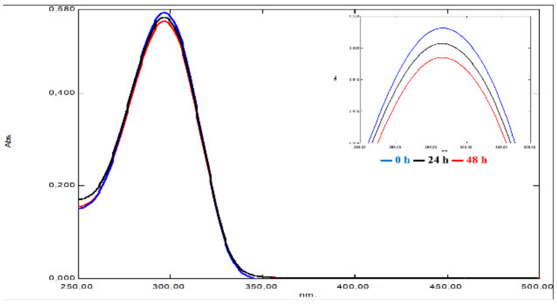

Complex 2

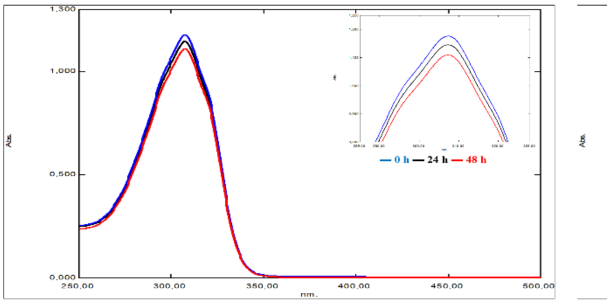

Complex 3

Figure S16. UV spectra of the complexes (**1-3**) for 0 h, 24 h and 48 h in PBS (10μM).

Table S1. Bond distances and bond angles of inter- and intramolecular hydrogen bonds forthe complexes (**1**–**3**).

D-H···A	d(D-H) Å	d(H···A) Å	d(D···A) Å	(DHA) deg.	Symmetry Codes
			(1)		
N7-H7···Cl3	0.860	2.4676	3.302(3)	163.6	
NC-HF···Cl3	0.861	2.8851	3.246(3)	107.3	1+x,y,z
NC-HG···Cl3	0.860	2.9496	3.246(3)	102.5	1+x,y,z
NB-HA···Cl5	0.861	2.7830	3.501(3)	142.0	-1+x,y,z
NC-HG···S2	0.860	2.5139	3.372(3)	176.4	-1+x,y,z
			(2)		
N31-H31···Cl1	0.88	2.371	3.20(1)	157.2	-1+x,y,z
N29-H29···Cl1	0.880	2.408	3.202(9)	150.3	-1+x,y,z
C27'-H27A···Cl1	0.98	2.791	3.74(1)	163.2	-1+x,y,z
N11-H11···Cl2	0.880	2.683	3.400(9)	139.4	x,1+y,z
C1-H1···Cl2	0.95	2.830	3.62(1)	141.4	x,1+y,z
C32-H32B···Cl3	0.98	2.772	3.58(1)	140.4	-1+x,y,z
C23-H23···Cl3	0.95	2.893	3.83(1)	167.5	x,y,-1+z
C1R-H1RA···S10	0.990	2.895	3.74(1)	143.6	-1+x,-1+y,-1+z
			(3)		
N9-H9…Cl1	0.86	2.401	3.26(2)	177	
N49-H49…Cl3	0.86	2.434	3.29(2)	175	
N51-H51…O1	0.86	2.08	2.82(2)	144	
N51-H51…C4AA	0.86	2.83	3.38(2)	123	
C25-H25…Cl2	0.93	2.827	3.74(1)	166.7	-1+x,y,z
N28-H28…Cl2	0.86	2.895	3.73(1)	164.0	-1+x,y,z
N31-H31…Cl2	0.86	2.466	3.22(1)	146.4	-1+x,y,z
C4-H4…S10	0.93	2.946	3.83(2)	159	x,y,-1+z

Table S2. Molar conductivity values of the complexes **1-4** for 0 h, 24 h and 48 h in DMSO ($\Omega^{-1}cm^2mol^{-1}$).

Compounds	0 h	24 h	48 h
**1**	21.60±0.65	25.10±0.43	26.40±0.28
**2**	22.50±0.85	24.76±0.57	26.53±0.29
**3**	5.80±0.25	6.40±0.29	7.13±0.21
